# Synthesis and Antimicrobial, Anticancer and Anti-Oxidant Activities of Novel 2,3-Dihydropyrido[2,3-d]pyrimidine-4-one and Pyrrolo[2,1-b][1,3]benzothiazole Derivatives via Microwave-Assisted Synthesis

**DOI:** 10.3390/molecules27041246

**Published:** 2022-02-12

**Authors:** Aamal A. Al-Mutairi, Hend N. Hafez, Abdel-Rhaman B. A. El-Gazzar, Marwa Y. A. Mohamed

**Affiliations:** 1 Chemistry Department, Faculty of Science, Saudi Arabia; Imam Mohammad Ibn Saud Islamic University (IMSIU), P.O. Box 90950, Riyadh 11623, Saudi Arabia; aamutairi@imamu.edu.sa; 2 Heterocyclic & Nucleosides Unit, Photochemistry Department, National Research Centre, Dokki, Giza 12622, Egypt; profelgazzar@yahoo.com; 3 Biology Department, Faculty of Science, Saudi Arabia; Imam Mohammad Ibn Saud Islamic University (IMSIU), P.O. Box 90950, Riyadh 11623, Saudi Arabia; Yousry_marwa@yahoo.com

**Keywords:** pyrido[2,3-d]pyrimidine, pyrrolo[2,1-b][1,3]benzothiazole, arylidenes, microwave, antimicrobial, anticancer, antioxidant activity

## Abstract

In our attempt towards the synthesis and development of effective antimicrobial, anticancer and antioxidant agents, a novel series of 2,3-dihydropyrido[2,3-d]pyrimidin-4-one **7a**–**e** and pyrrolo[2,1-b][1,3]benzothiazoles **9a**–**e** were synthesized. The synthesis of 2-(1,3-benzo thiazol-2-yl)-3-(aryl)prop-2-enenitrile (**5a**–**e**) as the key intermediate was accomplished by a microwave efficient method. Via a new variety oriented synthetic microwave pathway, these highly functionalized building blocks allowed access to numerous fused heteroaromatic such as 7-amino-6-(1,3-benzo thiazol-2-yl)-5-(aryl)-2-thioxo-2,3dihydropyrido [2,3-d]pyrimidin-4(1H)-one **7a**–**e** and 1-amino-2-(aryl)pyrrolo[2,1-b][1,3]benzothiazole-3-carbonitrile derivatives **9a**–**e** in order to study their antimicrobial and anticancer activity. The present investigation offers effective and rapid new procedures for the synthesis of the newly polycondensed heterocyclic ring systems. All the newly synthesized compounds were evaluated for antimicrobial, anticancer and antioxidant activity. Compounds **7a**,**d**, and **9a**,**d** showed higher antimicrobial activity than cefotaxime and fluconazole while the remaining compounds exhibited good to moderate activity against bacteria and fungi. An anticancer evaluation of the newly synthesized compounds against the three tumor cell lines (lung cell NCI-H460, liver cancer HepG2 and colon cancer HCT-116) exhibited that compounds **7a**, **d**, and **9a**,**d** have higher cytotoxicity against the three human cell lines compared to doxorubicin as a reference drug. These compounds also exhibited higher antioxidant activity and a great ability to protect DNA from damage induced by bleomycin.

## 1. Introduction

**Figure 1:**
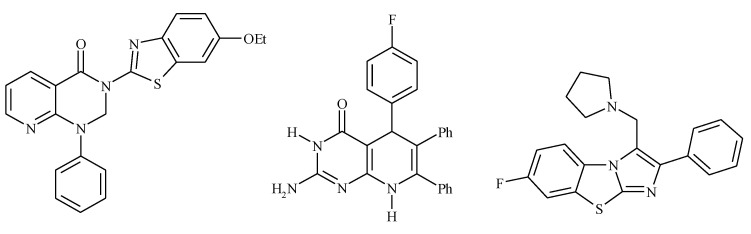
Chemical structure of biologically active compounds.

Cancer is one of the most serious health problems globally and is one of the main causes of increases in the death rate. Accordingly, there is an urgent requirement to make more effort to modify drugs and motivate the synthesis of new chemical bioactive compounds that establish improvement through current therapies. Pyrido[2,3-d]pyrimidine is one of the most privileged heterocyclic frameworks in several efficacious pharmacological compounds. Pyrido[2,3-d]pyrimidine ring systems have assorted biological and pharmacological activities such as being analgesic, anti-inflammatory [1,2,3], antitubercular [4], antimicrobial [5,6,7], a threonine tyrosine kinase (TTK) inhibitor [8], an adenosine kinase inhibitor [9], antiviral [10], antioxidant [11,12,13,14], a dihydrofolate reductase inhibitor [15,16], and an efficient glucosidase inhibitor [17,18]. Moreover, the 1,3-benzothiazole nucleus is a highly significant scaffold in the drug design, due to important pharmacological and medicinal activities, such as being antiviral [19], antituberculosis [20], an identifier of selective CB2 receptor ligands [21], antitumor [22,23,24], antimicrobial [25,26,27], anticonvulsant [28], a schistosome BCL-2 inhibitor [29], antidiabetic [30], antioxidant [31], an anti-Alzheimer drug [32] and a urease inhibitor [33] among the heterocyclic compounds containing a pyrimidine and benzothiazole nucleus that exhibits biological activity [34,35,36] (Figure 1).

Accordingly, with all of the previous observations of the biological importance and in continuation of our program in the synthesis of pyrido[2,3-d]pyrimidine [37], this study aims to design and develop highly selective and efficacious antimicrobial and anticancer agents of a novel series of pyrido[2,3-d]pyrimidine derivatives bearing different heterocyclic and aryl moieties such as benzothiazole, thiophene, furan, piperonal, naphthalene, and fluorophenyl as a side chain and various aryl derivatives by the microwave irradiation technique.

We have also reported here the synthesis of pyrrolo[2,1-b][1,3]benzothiazole bearing aryl and heteroaryl derivatives under microwave irradiation in the hope of obtaining novel antimicrobial and anticancer agents with excellent yield. Nowadays, the microwave irradiation method is a steady and attractive method for the synthesis of polycondensed heterocyclic compounds due to rapid, simple, and high yields [38,39]. 

## 2. Results

### 2.1. Chemistry 

**Scheme 1:**
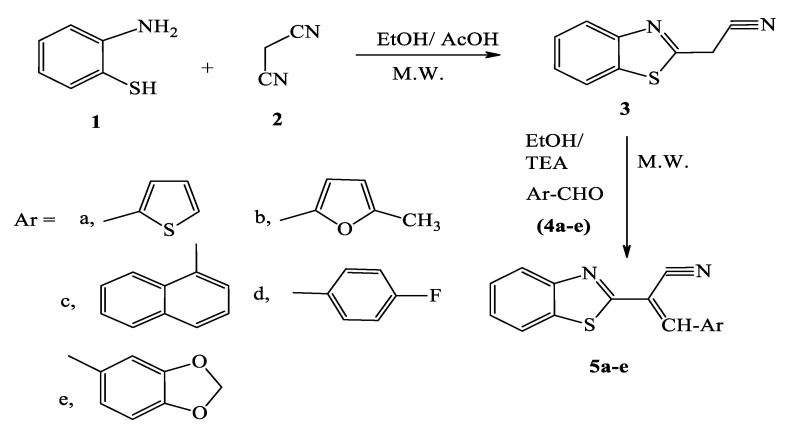
Synthesis of 2-(1,3-benzothiazol-2-yl)-3-(aryl)prop-2-enenitrile derivatives (**5a**–**e**).

The synthetic strategy of 2-(1,3-benzothiazol-2-yl)-3-(aryl)prop-2-enenitrile derivatives **5a**–**e** includes two steps outlined in Scheme 1. Synthesis of 2-cyano -methyl-1,3-benzothiazol **3** by a newly highly efficient method with high yield (92%) under microwave irradiation at 40 °C for 10 min by the reaction of 2-amino thiophenol **1** with malononitrile **2** in ethanol (10 mL) and acetic acid as a catalyst is compared to the old method with low yield and long reaction time [40]. Knoevenagel condensation of active methylene **3** with appropriate aromatic aldehyde using absolute ethanol in the presence of a catalytic amount of triethylamine under MWI at 60 °C afforded compounds **5a**–**e** as shown in Table 1. The progress of the reaction was followed by TLC (petroleum ether (80–90): ethylacetate, ratio: 3:1). The structure of the newly synthesized compounds was established by spectral data and elemental analysis.

Under microwave irradiation conditions, the treatment of the reactive intermediates **5a**–**e** with 6-aminothiouracil **6** using triethylamine as a catalyst in absolute ethanol at 100 °C generated the desired product **7a**–**e** with (49–53%) yield within 45 min. Interestingly, the use of dimethylformamide instead of ethanol as a solvent without a catalyst at 150 °C afforded the most satisfactory results of the corresponding 7-amino-(1,3-benzothiazol-2-yl)-2-thioxo-2,3-dihydropyrido[2,3-d]pyrimidin-4-one derivatives **7a**–**e** in high yield within 25 min as shown in (Table 2).

**Scheme 2:**
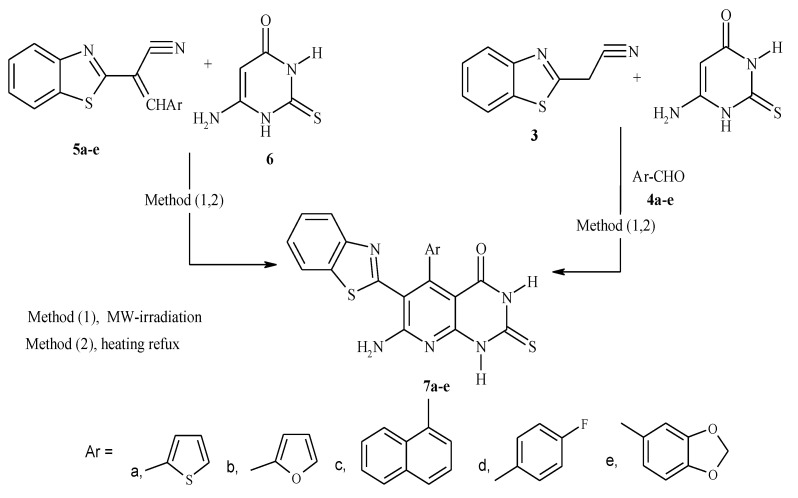
Synthesis of 5-amino-6-(1,3-benzothiazol-2-yl)-7-(substituted phenyl)-2-thioxo- 2,3-dihydro-pyrido[2,3-d]pyrimidin-4(1*H*)-one derivatives **7a**–**e**.

Nevertheless, heating under reflux of compounds **5a**–**e** with **6** whether in DMF or ethanol\TEA for a long time afforded (**7a**–**e**) as shown in Scheme 2. 

**Scheme 3:**
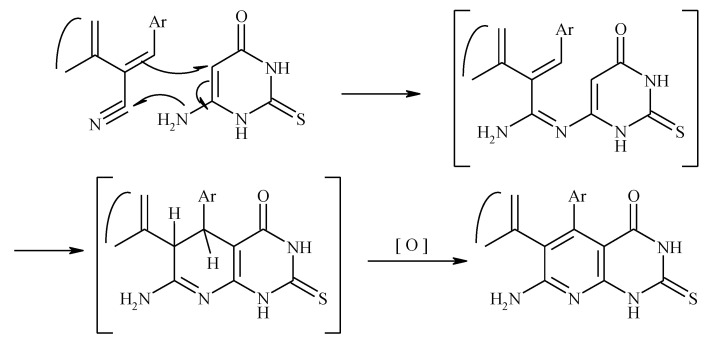
Mechanism for formation of 2,3-dihydropyrido[2,3-d]pyrimidin-4-one derivatives (**7a**–**e**).

A possible mechanism for the formation of 2,3-dihydropyrido[2,3-d]pyrimidin-4-one derivatives (**7a**–**e**) was proposed in Scheme 3. 

In this research, we have also studied the possibility of the multicomponent reaction of 2-cyanomethyl-1,3-benzothiazol **3**, 6-aminothiouracil **6**, and appropriate aldehydes **4a**–**e** under microwave irradiation conditions at 150 °C and TLC control. Pyrido[2,3-d]pyrimidine derivatives **7a**–**e** were accumulated, the reaction condition has been established in Table 3. On the other hand, the same reaction was carried out by conventional heating under reflux in DMF as shown in Scheme 2. 

**Scheme 4:**
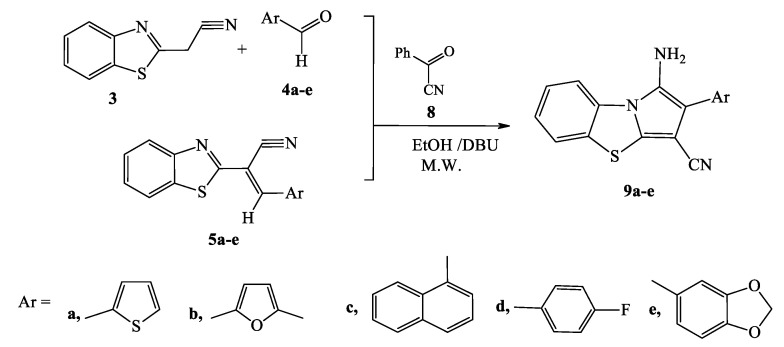
Synthesis of 1-amino-pyrrolobenzothiazole-3-carbonitrile derivatives (**9a**–**e**) under microwave as assisted method.

Furthermore, in our progression in the development of the highly efficient method in the synthesis of newly fused heterocyclic compounds with expected biological activity. we report the synthesis of novel pyrrolo[2,1-b][1,3]benzothiazole derivatives **9a**–**e,** Scheme 4, via microwave-assisted three-component reactions and the evaluation of their cytotoxicity, leading to the discovery of some new heterocycles with potent cytotoxic activity higher than or similar to doxorubicin as standard drug.

Initially, under microwave irradiation of three-component of 2-cyanomethyl -1,3-benzothiazol **3**, benzoyl cyanide **8** and, appropriate aldehyde such as 4-fluorobenzaldhyde **4d** was employed to optimize the reaction conditions as shown in (Table 4). Firstly, (MWI, 100 °C, 25 min) solvent screening showed that the usage of methanol or ethanol as polar solvents was beneficial while in dichloroethane no reaction occurred (entry 2). The presence of bases also increases the yield percentage as shown in Table 4. DBU was shown to be an effective base catalyst more than TEA. So, EtOH/DBU was preferred as the optimal solvent/catalyst system and 120 °C was selected as the most convenient reaction temperature (Table 4, entry 7) in view of the highest yield of **9d** (87%)**.** Under the optimized reaction conditions, various aldehydes **4a**–**e** were treated with 2-cyanomethyl-1,3-benzothiazol **3** and benzoyl cyanide **8,** as illustrated in Table 4, while treatment of the three components under conventional heating took a long time and afforded a moderate yield.

**Scheme 5:**
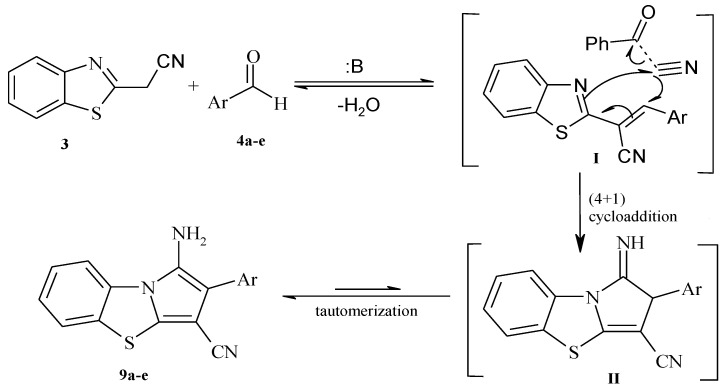
Synthetic mechanism of pyrrolobenzothiazole-3-carbonitrile derivatives **9a**–**e**.

A reasonable reaction mechanism for the synthesis of 1-amino pyrrolobenzothiazole-3-carbonitrile derivatives **9a**–**e** is shown in Scheme 5. The reaction involves a base-catalyzed two-step formation of 2-arylidene cyanomethyl 1,3-benzothiazoles via a Knoevenagel condensation between 2-cyanomethyl-1,3-benzothiazol and aldehydes, followed by [4+1] cycloaddition of benzoyl cyanide.

Finally, as illustrated in Table 5, the corresponding products **9a**–**e** were successfully achieved in high yield compared with the three-component reaction through the treatment of the reactive intermediates **5a**–**e** with benzoyl cyanide **8** under microwave irradiation at 120 °C for 20–25 min in EtOH/DBU, while conventional heating in ethanol/TEA took a long time. As is evident, the yield afforded from the treatment of **5a**–**e** with benzoyl cyanide **8** was the best, compared to the yield from multicomponent reactions under microwave irradiation. So, it is now settled that microwaves can greatly speed up reactions and improve overall yield.

### 2.2. Biological Activities

#### 2.2.1. Antimicrobial Activity and Structure Activity Relationship

The values of *MIC* of the synthesized compounds against the tested microorganisms are displayed in Table 6 and Table 7. Benzothiazole arylidine derivatives **5a**–**e** exhibited good to moderate antimicrobial activities. These derivatives increased the antimicrobial activity when treated with 6-aminothiouracil to afford 2,3-dihydropyrido [2,3-d] pyrimidine-4-one derivatives. The investigations showed significant inhibitory effects against bacteria with the majority of the compounds with *MIC* values of (4–20 μmol L^–1^) (Table 6). It was found that compounds **7a**,**d** were found to be more potent against bacteria with *MIC* value ranging from (4–12 μmol L^–1^) than cefotaxime with *MIC* value (6–12 μmol L^–1^), while compounds **7b**, **7c** and **7e** exhibited good activity against all the bacterial strains. This high efficacy may be attributed to the presence of a benzothiazol and thiophene moiety as compound **7a** and the presence of an electron withdrawing group (fluoro) in the para-position of the phenyl ring attached to the pyridopyrimidine backbone **7d**. Compound **7a** had equipotent activity with *MIC* (6, 12 μmol L^–1^) as cefotaxime against Bacillus *subtilis* and *Chlamydia pneumonia,* respectively. Compound **7e** was also equipotent with *MIC* (8 μmol L^–1^) as cefotaxime against *Salmonella typhi*. Pyrrolo[2,1-b][1,3]benzothiazole derivatives **9a**–**e** could effectively inhibit the growth of the tested bacteria strains. Compound **9a** was more potent against *Staphylococcus aureus* while it had equipotent efficacy against *Bacillus subtilis* and *Chlamydia pneumonia* to cefotaxime; this was attributed to the presence of pyrrolo [2,1-b][1,3]benzothiazole with thiophene moiety as a side chain. Compound **9d** with *MIC* (4–10 μmol L^–1^) had exceptional activity toward all bacteria due to the presence of pyrrolobenzothiazole with *p*-fluorophenyl substituent which increased the antibacterial potency compared to the reference drug. On the other hand, compounds **9b**, **9c** and **9e** exhibited good inhibition activity against all bacterial strains (Table 6).

The newly synthesized compounds were evaluated for their antifungal activity against three fungal strains displayed in (Table 7). Most of the synthesized compounds exhibited good to moderate inhibition activities against all the fungal strains. Compound **9d** exhibited more potent activity than fluconazole against *Candida albicans* and *Ganoderma lucidum* while it had equipotent activity against *Aspergillus flavus.* Compound **9a** exhibited stronger activity than fluconazole against *Candida albicans and* excellent activity against *Aspergillus flavus* and *Ganoderma lucidum.* Compound **9b** had good inhibition activity against *Aspergillus flavus* and *Candida albicans* but moderate activity toward *Ganoderma lucidum.* Compound **9e** also had promising activity against all fungal strains.

Compounds **7a**,**d** had good inhibition activity against all fungus, while compounds **5a**–**e**, **7b**, **7c** and **7e** exhibited good to moderate activities. The introduction of benzothiazole, thiophene, and p-fluorophenyl moieties to the pyridopyrimidine derivatives and the presence of pyrrolobenzothiazole derivatives with thiophene and *p*-fluorophenyl side chains might be responsible for the antifungal activity enhancement of these compounds.

#### 2.2.2. Cytotoxicity Screening and Structure Activity Relationship (SAR)

The cytotoxic activity of all the newly synthesized compounds benzothiazole arylidine **5a**–**e**, pyrido[2,3-d]pyrimidine **7a**–**e** and pyrrolo[2,1-b][1,3] benzothiazole derivatives **9a**–**e** were evaluated against three tumor cell lines (human lung cell NCI-H460, liver cancer HepG2 and colon cancer HCT-116) by MTT assay. The three human cancer cell lines were provided by the National Cancer Institute (NCI, Cairo, Egypt). Doxorubicin was used as the positive control. The cytotoxic activities are expressed as the median growth inhibitory concentration (IC_50_) and are provided in (Table 8). From the results, it is evident that most of the newly synthesized compounds showed potent to moderate cytotoxic activity against the three tumor cell lines. The results of the series of compound **5** indicated that the type of the side chain on the benzothiazole arylidine derivatives occupied significant roles in the cytotoxic activity. Compounds **5a** with IC_50_ values of (3.61, 3.14 and 4.20 μmol L^−1^) and **5d** with IC_50_ values of (3.04, 3.20 and 3.38 μmol L^−1^) showed good antitumor activity toward all tumor cell lines, while the other compounds **5b**, **5c** and **5e** exhibited moderate activity in the range of (IC_50_ = 5.82–10.20 μmol L^−1^) compared to doxorubicin.

The series of 2,3-dihydropyrido[2,3-d]pyrimidine-4-one derivatives **7a**–**e** exhibited higher cytotoxic activity than the series of pyrrolo [2,1-b][1,3] benzothiazole **9a**–**e**. Table 8 shows 2,3-dihydropyrido[2,3-d]pyrimidine-4-one **7a** containing benzothiazol and thiophene as a biologically active side chain with IC_50_ values of (0.66, 0.45 and 0.70 μmol L^−1^), benzothiazol and p-fluorophenyl moieties **7d** with IC_50_ values of (0.28, 0.39 and 0.14 μmol L^−1^), pyrrolo[2,1-b][1,3]benzothiazole **9a** with benzothiazol and thiophene with IC_50_ values of (1.10, 0.49 and 0.89 μmol L^−1^), benzothiazol and p-fluorophenyl **9d** side chain moieties with IC_50_ values of (0.45, 0.47 and 0.59 μmol L^−1^) were found to be more potent and efficacious than the reference drug doxorubicin with IC_50_ values of (1.98, 0.52 and 1.12 μmol L^−1^). It was noticeable that the presence of side chain *p*-fluorophenyl improved the antitumor activity more than thiophene side chain in addition to the basic skeleton.

In addition, 5-methylfuranyl **7b** with IC_50_ values of (2.56, 2.89, 2.68), 5-piperonyl- 2,3-dihydropyrido[2,3-d]pyrimidine-4-one and **7e** (2.60, 3.00 and 3.08 μmol L^−1^) exhibited good cytotoxic effect towards the three tumor cell lines. On the other hand, 5-methylfuranyl **9b** with IC_50_ (3. 20, 3.54 and 3.89 μmol L^−1^) and 2-piperonyl pyrrolo[2,1-b][1,3]benzothiazole **9e** with *IC50* (3.90, 4.08 and 4.50 μmol L^−1^) had good antitumor activity against all tumor cell lines. Compounds with 5-naphthalenyl side chain in both 2,3-dihydropyrido[2,3-d]pyrimidine-4-one **7c** and pyrrolo[2,1-b][1,2]benzothiazole **9c** showed moderate antitumor activity. From the above structure activity relationships (SAR), the most selective 2,3-dihydropyrido[2,3-d]pyrimidine-4-one and pyrrolo [2,1-b] [1,3]benzothiazole on the studied cancer cell lines were bearing p-fluorophenyl and thiophene moieties. Thus, among the promising candidates against cancer cell lines compounds **7a**, **7d**, **9a**, and **9d** ended up being the best pharmacophores for developing a new drug candidate for cancer.

Generally, the type of substituent plays an important role in antitumor activity and the presence of the basic skeleton of fused heterocyclic compounds improves the cytotoxic activity in cancer cells. 

#### 2.2.3. Antioxidant Activity Screening Assay

A series of 2,3-dihydropyrido[2,3-d]pyrimidine-4-one **7a**–**e** and pyrrolo[2,1-b][1,3] benzothiazole **9a**–**e** derivatives were tested for antioxidant activity as reflected in the ability to inhibit lipid peroxidation in rat brain and kidney homogenates by using (3-ethylbenzthiazoline-6-sulfonic acid) ABTS free radical scavenging, see Table 9. Compounds **7a** (91.2%), **7d** (92.8%) and **9d** (90.4%) exhibited potent inhibition higher than Trolox (89.5%), while compound **9a** (88.7%) showed nearly equipotent inhibition activity. On the other hand, the remaining compounds **7b**, **7c**, **7e**, **9b**, **9c** and **9e** showed good antioxidant activities Table 9.

#### 2.2.4. Bleomycin-Dependent DNA Damage

The pro-oxidant activities of compounds **7a**–**e** and **9a**–**e** were assessed by their effects on bleomycin-induced DNA damage. Analysis of the data in Table 9 showed that, compounds **7a** (0.053), **7d** (0.045), and **9d** (0.064) were found to be more potent than the reference drug Trolox (0.067). Moreover, compound **9a** (0.069) had almost equipotent activity, that means these compounds have great ability to protect DNA from the damage induced by bleomycin. The rest of compounds **7b**, **7c**, **7e**, **9b**, **9c** and **9e** exhibited excellent protection against DNA damage.

## 3. Materials and Methods

### 3.1. Reagents and Materials

Melting points were recorded on an advanced melting point apparatus, SMP30, Stuart (Bibby Scientific, London, UK). Microanalytical data were gathered with a Vario Elementar apparatus (Shimadzu-Japan). Elemental analyses of all compounds were within ± 0.4% of the theoretical values. The IR spectra (KBr) were recorded on a Perkin Elmer 1650 spectrometer (USA). ^1^H and ^13^C NMR spectra (500 MHz for ^1^H and 125 MHz for ^13^C NMR) were recorded on JEOL ECA-500 (Shimadzu) instruments.

Chemical shifts were expressed in ppm relative to SiMe_4_ as an internal standard in DMSO-*d_6_* or (CDCl_3_) as a solvent. Mass spectra were recorded on a 70 eV Finnigan SSQ 7000 spectrometer (Thermo-Instrument System Incorporation, USA). Follow up of the reaction and the purity of the compounds was checked on aluminum plates coated with silica gel (Merck, Germany) on Microwave Advanced Flexible Synthesis Platform 1900 W (flexiWAVE—Milestone, Italy), producing continuous irradiation and equipped with a simultaneous external air-cooling system. Chemicals and solvents (Analar ≥ 99%) were purchased from Sigma-Aldrich (USA). 

### 3.2. Syntheses

2-(1,3-benzothiazol-2-yl)-3-(aryl)prop-2-enenitrile (**5a**–**e**).

General procedure. A mixture of compound **3** (0.87 g, 5 mmol), (5 mmol) of the appropriate aromatic aldehyde (thiophene-2-carboxaldehyde, 5-methylfuran-carbox- aldhyde, 1-naphthaldhyde, 4-fluorobenzaldhyde, and piperonal) **4a**–**e** and TEA in ethanol was subjected to MWI at 60 °C for 4–8 min. The progress of the reaction was followed by TLC (petroleum ether (80–90): ethyl acetate, ratio:3:1). After cooling, solids were obtained, filtered, and recrystallized from a proper solvent to give **5a**–**e**, respectively.

2-(1,3-benzothiazol-2-yl)-3-(thiophen-2-yl)prop-2-enenitrile (**5a**).

Green powder, crystallized from cyclohexane, mp. 153–155 °C; IR (KBr, cm^−1^); 3108 (CH aryl), 2212 (CN), 1600 (C=N); ^1^H NMR (500 MHz, CDCl_3_, *δ*, ppm); 7.25(s, 1H, thiophene), 7.42 (t, 1H, Ar-*H*), 7.52 (t, 1H, Ar-*H*), 7.71 (d, 1H, *J* = 4.92 Hz, thiophene), 7.82 (d, 1H, *J* = 5.1 Hz, thiophene), 7.91 (d, 1H, *J* = 8.01 Hz, Ar-*H*), 8.05 (d, 1H, *J* = 8.01 Hz, Ar-*H*), 8.40 (s, 1H, =CH),; Its MS (m/z), 268 (M^+^); C_14_H_8_N_2_S_2_ (268.3); Anal. Calcd.; % C: 62.66, % H: 3.00, % N: 10.44; found % C: 62.64, % H: 2.98, % N: 10.41. (see Supporting Information).

2-(1,3-benzothiazol-2-yl)-3-(5-methylfuran-2-yl)prop-2-enenitrile (**5b**).

Brown powder, crystallized from ethanol, mp. 150–151 °C; IR (KBr, cm^−1^); 2934, 2899 (CH aliphatic), 3054 (CH aryl), 2226 (CN), 1588 (C=N); ^1^H NMR (500 MHz, CDCl_3_, *δ*, ppm); 2.44 (s, 3H, CH_3_), 6.27(d, 1H, *J* = 5.6 Hz, furan), 7.19 (d, 1H, *J* = 5.68 Hz, furan), 7.44 (t, 1H, Ar-*H*), 7.48 (t, 1H, Ar-*H*), 7.84 (d, 1H, *J* = 8.10 Hz, Ar-*H*), 7.92 (s, 1H, =CH), 8.00 (d, 1H, *J* = 8.10 Hz, Ar-*H*); Its MS (m/z), 266 (M^+^); C_15_H_10_N_2_OS (266.3); Anal. Calcd.; % C: 67.65, % H: 3.78, % N: 10.52; found % C: 67.63, % H: 3.75, % N: 10.49.

2-(1,3-benzothiazol-2-yl)-3-(naphthalen-1-yl)prop-2-enenitrile (**5c**).

Pale yellow powder, crystallized from dioxane, mp. 128–130 °C; IR (KBr, cm^−1^); 3050 (CH aryl), 2218 (CN), 1587 (C=N); ^1^H NMR (500 MHz, CDCl_3_, *δ*, ppm); 7.61 (t, 1H, Ar-*H*),7.80 (m, 4H, Ar-*H*), 8.02 (m, 2H, Ar-*H*), 8.11 (d, 1H, *J* = 8.76 Hz, Ar-*H*), 8.12 (m, 2H, Ar-*H*), 8.14 (d, 1H, *J* = 8.76 Hz, Ar-*H*), 9.08 (s, 1H, =CH); Its MS (m/z), 312 (M^+^, 80%); C_20_H_12_N_2_S (312.3); Anal. Calcd.; % C: 76.90, % H: 3.87, % N: 8.97; found % C: 76.88, % H: 3.85, % N: 8.94.

2-(1,3-benzothiazol-2-yl)-3-(4-fluorophenyl)prop-2-enenitrile (**5d**).

Yellow powder, crystallized from ethanol, mp. 161–163 °C; IR (KBr, cm^−1^); 3054 (CH aryl), 2226 (CN), 1588 (C=N); ^1^H NMR (500 MHz, CDCl_3_, *δ*, ppm); 7.20 (m, 2H, Ar-*H*), 7.42 (t, 1H, Ar-*H*), 7.50 (t, 1H, Ar-*H*), 7.90 (d, 1H, *J* = 8.10 Hz, Ar-*H*), 8.04 (m, 3H, Ar-*H*), 8.19 (s, 1H, =CH); Its MS (m/z), 280 (M^+^, 73%); C_16_H_9_FN_2_S (280.3); Anal. Calcd.; % C: 68.55, % H: 3.24, % N: 9.99; found % C: 68.54, % H: 3.22, % N: 9.97.

2-(1,3-benzothiazol-2-yl)-3-(piperon-2-yl)prop-2-enenitrile (**5e**).

Yellow powder, crystallized from dioxane, mp. 222–224 °C; IR (KBr, cm^−1^); 2938, 2874 (CH aliphatic), 3054 (CH aryl), 2223 (CN), 1587 (C=N); ^1^H NMR (500 MHz, CDCl_3_, *δ*, ppm); 6.08 (s, 2H, O-CH_2_-O), 6.91 (d, 1H, *J* = 7.05 Hz, Ar-*H*), 7.41–7.45 (m, 2H, Ar-*H*), 7.50–7.51 (t, 1H, Ar-*H*), 7.73 (s, 1H, Ar-*H*), 7.89 (d, 1H, *J* = 6.9 Hz, Ar-*H*), 8.05 (d, 1H, *J* = 6.9 Hz, Ar-*H*), 8.13 (s, 1H, =CH); Its MS (m/z), 306 (M^+^, 66%); C_17_H_10_N_2_O_2_S (306.3); Anal. Calcd.; % C: 66.64, % H: 3.92, % N: 9.14; found % C: 66.62, % H: 3.90, % N: 9.13.

7-amino-6-(1,3-benzothiazol-2-yl)-5-(aryl)-2-thioxo-2,3dihydropyrido[2,3-d]pyrimidin-4(1H)-one (**7a**–**e**).

General procedure: (Method 1) **under microwave irradiation**.

(Method 1a): A mixture of compounds **5a**–**e** (5 mmol) and 6-aminothiouracil **6** (0.7 g, 5 mmol) in DMF was placed in a microwave process vial. The vial was closed and subjected to microwave irradiation for 25 min at 150 °C/or in EtOH/TEA for 45 min at 100 °C. The progress of the reaction was followed by TLC. After cooling to room temperature, the solid formed was filtered off, washed with ethanol, dried, and crystallized from DMF.

(Method 1b): A mixture of 2-cyanomethyl-1,3-benzothiazol **3** (0.87 g, 5 mmol)**,** 6-amino thiouracil **6** (0.7 g, 5 mmol), and appropriate aldehydes **4a**–**e** (5 mmol) in DMF was placed in a microwave process vial. The vial was closed and subjected to microwave irradiation for 50 min at 150 °C. The progress of the reaction was followed by TLC. After cooling to room temperature, the solid formed was filtered off, washed with ethanol, dried, and crystallized from DMF.

(Method 2) **using conventional heating**.

(Method 2a): A mixture of compounds **5a**–**e** (10 mmol) and 6-aminothiouracil **6** (1.43 g, 10 mmol) was heated under reflux in DMF/or EtOH/TEA for several hours. The reaction mixture was allowed to cool to room temperature and the solid formed was filtered off, dried, and crystallized from DMF.

(Method 2b): A mixture of 2-cyanomethyl-1,3-benzothiazol **3** (1.74 g, 10 mmol)**,** 6-amino thiouracil **6** (1.43 g, 10 mmol), and appropriate aldehydes **4a**–**e** (10 mmol) was heated under reflux in DMF for several hours. The reaction mixture was allowed to cool to room temperature and the solid formed was filtered off, dried, and crystallized from DMF.

7-amino-6-(1,3-benzothiazol-2-yl)-5-(thiophen-2-yl)-2-thioxo-2,3-dihydropyrido[2,3-d]pyrimidin-4(1H)-one (**7a**).

Brown powder, mp. > 300 °C; IR (KBr, cm^−1^); 3379, 3273 (NH_2_, NH), 3074 (CH aryl), 1680 (C=O), 1599 (C=N); ^1^H NMR (500 MHz, DMSO-*d*_6_, δ, ppm); 3.32–3.53 (brs, 1H, NH, D_2_O exchangeable), 7.19–7.21(m, 2H, Ar-*H*), 7.23 (m, 1H, Ar-*H*), 7.44 (brs, 1H, Ar-*H*), 7.45 (brs, 1H, Ar-*H*), 7.46 (brs, 1H, Ar-*H*), 7.59 (brs, 1H, Ar-*H*), 8.10 (d, 1H, *J* = 7.45 Hz, Ar-*H*), 12.31 (brs, 1H, NH), 13.00 (brs, 1H, NH) (NH_2_, 2NH,D_2_O exchangeable); ^13^C-NMR (125 MHz, DMSO-*d*_6_) δ ppm: 108.10, 114.00, 115.20, 118.00, 129.00, 130.00, 131.10, 131.50, 132.10, 135.80, 142.10, 149.20, 150.00, 156.80, 159.80, 160.00, 162.00 (C=O), 175.00 (C=S); Its MS (m/z), 409 (M^+^, 74%); C_18_H_11_N_5_OS_3_ (409.5); Anal. Calcd.; % C: 52.79, % H: 2.71, % N: 17.10; found % C: 52.76, % H: 2.70, % N: 17.08.

7-amino-6-(1,3-benzothiazol-2-yl)-5-(5-methylfuran-2-yl)-2-thioxo-2,3-dihydropyrido[2,3-d]pyrimidin-4(1H)-one (**7b**).

Pale red powder, mp. > 300 °C; IR (KBr, cm^−1^); 3400, 3381 (NH_2_, NH), 3045 (CH aryl), 2938 (CH aliphatic), 1675 (C=O), 1608 (C=N); ^1^H NMR (500 MHz, DMSO-*d*_6_, δ, ppm); 2.85 (s, 3H, CH_3_), 3.32 (brs, 2H, NH_2_ + H_2_O), 7.20 (d, 1H, *J* = 6.01 Hz, furan), 7.36–7.44 (m, 4H, Ar-*H*), 7.93 (d, 1H, *J* = 6.01 Hz, furan), 12.33, 13.07 (2brs, 2NH) (NH_2_, 2NH, D_2_O exchangeable); ^13^C-NMR (125 MHz, DMSO-*d*_6_) δ ppm: 19.50, 108.00, 114.10, 115.00, 118.80, 129.00, 130.00, 131.10, 131.40, 132.00, 135.80, 143.00, 149.20, 153.00, 156.80, 159.80, 162.00, 164.00 (C=O), 176.00 (C=S ); Its MS (m/z), 407 (M^+^, 70%); C_19_H_13_N_5_O_2_S_2_ (407.4); Anal. Calcd.; % C: 56.01, % H: 3.22, % N: 17.19; found % C: 56.00, % H: 3.19, % N: 17.17.

7-amino-6-(1,3-benzothiazol-2-yl)-5-(naphthalen-1-yl)-2-thioxo-2,3-dihydro pyrido[2,3-d]pyrimidin-4(1H)-one (**7c**).

Yellow powder, mp. > 300 °C; IR (KBr, cm^−1^); 3373, 3342 (NH_2_, NH), 3061 (CH aryl), 1678 (C=O), 1597 (C=N); ^1^H NMR (500 MHz, DMSO-*d*_6_, δ, ppm); 3.38 (brs, 2H, NH_2_), 7.29–7.93 (m, 11H, Ar-H), 11.88 (brs, 1H, NH), 12.76 (brs, 1H, NH) (NH_2_, 2NH, D_2_O exchangeable); ^13^C-NMR (125 MHz, DMSO-*d*_6_) δ ppm: 101.61, 110.42, 121.63, 122.90, 125.50, 126.04, 126.39, 126.65, 126.96, 127.46, 128.20, 129.00, 132.00, 133.90, 135.40, 135.90, 152.06, 153.41, 158.16, 160.10, 164.00 (C=O), 176.03 (C=S); Its MS (m/z), 453 (M^+^, 66%); C_24_H_15_N_5_OS_2_ (453.5); Anal. Calcd.; % C: 63.56, % H: 3.33, % N: 15.44; found % C: 63.53, % H: 3.31, % N: 15.40.

7-amino-6-(1,3-benzothiazol-2-yl)-5-(4-fluorophenyl)-2-thioxo-2,3-dihydropyrido[2,3-d] pyrimidin-4(1H)-one (**7d**).

Orange powder, mp. > 300 °C; IR (KBr, cm^−1^); 3380, 3368 (NH_2_, NH), 3059 (CH aryl), 1680 (C=O), 1600 (C=N); ^1^H NMR (500 MHz, DMSO-*d*_6_, δ, ppm); 3.05 (br, 2H, NH_2_), 6.82 (d, 2H, *J* = 7.9 Hz, Ar-*H,*), 7.10–7.99 (m, 4H, Ar-*H*), 8.11 (d, 2H, *J* = 8.1 Hz, Ar-*H*), 11.99, 12.69 (brs, 2H, 2NH) (NH_2_, 2NH, D_2_O exchangeable); ^13^C-NMR (125 MHz, DMSO-d_6_) δ ppm: 112.33, 115.51, 121.97, 122.67, 122.91, 123.16, 125.90, 126.70, 127.34, 131.88, 131.95, 133.41, 148.24, 152.20, 153.57, 153.74, 158.60, 159.71, 164.20 (C=O), 175.99 (C=S); Its MS (m/z), 421 (M^+^, 89%); C_20_H_12_FN_5_OS_2_ (421.4); Anal. Calcd.; % C: 56.99, % H: 2.87, % N: 16.62; found % C: 56.97, % H: 2.84, % N: 16.60.

7-amino-5-(piperon-2-yl)-6-(1,3-benzothiazol-2-yl)-2-thioxo-2,3-dihydropyrido[2,3-d]pyrimidin-4(1H)-one (**7e**).

Pale yellow powder, mp. > 300 °C; IR (KBr, cm^−1^); 3370, 3278 (NH_2_, NH), 3040 (CH aryl), 2924 (CH aliphatic), 1678 (C=O), 1608 (C=N); ^1^H NMR (500 MHz, DMSO-*d*_6_, δ, ppm); 6.08 (s, 2H, O-CH_2_-O), 6.90 (d, 1H, *J =* 6.9 Hz, Ar-*H*), 7.41–7.45 (m, 2H, Ar-*H*), 7.50–7.51 (m, 1H, Ar-*H*), 7.73 (s, 1H, Ar-*H*), 7.88 (d, 1H, *J =* 6.9 Hz, Ar-*H*), 8.05 (d, 1H, *J =* 7.01 Hz, Ar-*H*), 8.12, 9.63 and 9.73 (brs, 4H, NH_2_ and 2NH), (NH_2_, 2NH,D_2_O exchangeable); ^13^C-NMR (125 MHz, DMSO-d_6_) δ ppm: 102.10 (O-CH_2_-O), 108.00, 115.00, 118.20, 123.00, 128.00, 130.00, 132.10, 136.00, 142.00, 133.41, 153.00, 153.20, 156.40, 158.00, 162.00, 165.00, 166.00 (C=O), 175.99 (C=S); Its MS (m/z), 446 (M^+^, 71%); C_21_H_13_N_5_O_3_S_2_ (447.4); Anal. Calcd.; % C: 56.36, % H: 2.93, % N: 15.65; found % C: 56.33, % H: 2.92, % N: 15.64.

1-amino-2-(aryl)pyrrolo[2,1-b][1,3]benzothiazole-3-carbonitrile (**9a**–**e**).

General procedure: (Method 1) **under microwave irradiation**.

(Method 1a): A mixture of 2-cyanomethyl-1,3-benzothiazol **3** (0.87 g, 5 mmol), benzoyl cyanide **8** (0.65 g, 5 mmol) and appropriate aldehyde **4a**–**e** (5 mmol) in EtOH/DBU was placed in a microwave process vial. The vial was closed and subjected to microwave irradiation for 25 min at 120 °C. Cooling the mixture to room temperature the solid precipitate was collected by filtration, dried and crystallized from dioxane. 

(Method 1b): A mixture of compounds **5a**–**e** and benzoyl cyanide **8** in EtOH/DBU was placed in a microwave process vial. The vial was closed and subjected to microwave irradiation for (20–25) min at 120 °C. Cooling the mixture to room temperature, the solid precipitate was collected by filtration, dried and crystallized from dioxane.

(Method 2) **using conventional heating**.

(Method 2a): A mixture of 2-cyanomethyl-1,3-benzothiazol **3** (5 mmol), benzoyl cyanide **8** (5 mmol) and appropriate aldehyde **4a**–**e** was heated under reflux in ethanol and trimethylamine for an appropriate time. The reaction mixture was allowed to cool to room temperature and the solid formed was filtered off, dried and crystallized from dioxane.

(Method 2b): A mixture of compounds **5a**–**e** (5 mmol) and benzoyl cyanide **8** (5 mmol) was heated under reflux in ethanol and trimethylamine for an appropriate time. The reaction mixture was allowed to cool to room temperature and the solid formed was filtered off, dried and crystallized from dioxane.

1-amino-2-(thiophen-2-yl)pyrrolo[2,1-b][1,3]benzothiazole-3-carbonitrile (**9a**).

Deep yellow powder, mp. 255–257 °C; IR (KBr, cm^−1^); 3420, 3412 (NH_2_), 3068 (CH aryl), 2249 (CN); ^1^H NMR (500 MHz, DMSO-*d*_6_, δ, ppm); 7.19 (s, 1H, Ar-*H*), 7.38–7.69 (m, 2H, Ar-*H*), 7.78–7.85 (m, 2H, Ar-*H*), 7.87 (m, 1H, Ar-*H*), 8.02 (m, 1H, Ar-*H*), 8.34 (brs, NH, D_2_O exchangeable); ^13^C-NMR (125 MHz, DMSO-*d*_6_) δ ppm: 102.20 (CN), 121.74, 123.43, 125.88, 127.00, 128.57, 133.35, 135.08, 135.53, 136.97, 138.83, 153.74, 162.38. Its MS (m/z), 295 (M^+^, 90%); C_15_H_9_N_3_S_2_ (295.3); Anal. Calcd.; % C: 60.99, % H: 3.07, % N: 14.23; found % C: 60.97, % H: 3.07, % N: 14.21.

1-amino-2-(5-methylfuran-2-yl)pyrrolo[2,1-b][1,3]benzothiazole-3-carbonitrile (**9b**).

Brown powder, mp. 232–234 °C; IR (KBr, cm^−1^); 3415, 3409 (NH_2_), 3090 (CH aryl), 2233 (CN); ^1^H NMR (500 MHz, DMSO-*d*_6_, δ, ppm); 2.85 (s, 3H, CH_3_), 7.20 (d, 1H, *J =* 5.9 Hz, furan), 7.22 -7.44 (m, 4H, Ar-H), 7.93 (d, 1H, *J =* 5.9 Hz, furan), 12.33 (d, NH, D_2_O exchangeable); ^13^C-NMR (125 MHz, DMSO-*d*_6_) δ ppm: 22.50 (CH_3_), 92.32 (CN), 100.00, 102.99, 115.84, 128.36, 130.03, 133.57, 136.77, 158.67, 159.08, 159.44, 161.71. Its MS (m/z), 293 (M^+^, 94%); C_16_H_11_N_3_OS (293.3); Anal. Calcd.; % C: 65.51, % H: 3.78, % N: 14.32; found % C: 65.50, % H: 3.76, % N: 14.29.

1-amino-2-(naphthalen-1-yl)pyrrolo[2,1-b][1,3]benzothiazole-3-carbonitrile (**9c**).

Pale red powder, mp. 260–262 °C; IR (KBr, cm^−1^); 3430, 3411 (NH_2_), 3100 (CH aryl), 2235(CN); ^1^H NMR (500 MHz, DMSO-*d*_6_, δ, ppm); 7.45–7.58 (m, 5H, Ar-*H*), 7.91 (m, 2H, Ar-*H*), 7.99 (m, 2H, Ar-*H*), 8.12 (m, 1H, Ar-*H*) 8.30 (m, 1H, Ar-*H*), 9.03 (brs, NH, D_2_O exchangeable); ^13^C-NMR (125 MHz, DMSO-*d*_6_) δ ppm: 109.00 (CN), 121.83, 123.26, 123.88, 125.61, 126.23, 126.84, 127.10, 127.66, 127.82, 129.22, 129.52, 132.55, 144.78, 153.75, 162.45. Its MS (m/z), 339 (M^+^, 81%); C_21_H_13_N_3_S (339.4); Anal. Calcd.; % C: 74.31, % H: 3.86, % N: 12.38; found % C: 74.30, % H: 3.84, % N: 12.36.

1-amino-2-(4-fluorophenyl)pyrrolo[2,1-b][1,3]benzothiazole-3-carbonitrile (**9d**)**.**

Yellow powder, mp. 269–270 °C; IR (KBr, cm^−1^); 3439, 3418 (NH_2_), 3098 (CH aryl), 2250(CN); ^1^H NMR (500 MHz, DMSO-*d*_6_, δ, ppm); 7.25 (d, 2H, Ar-*H*, *J = 8.00*), 7.40–7.62 (m, 2H, Ar-*H*), 7.85 (d, 2H, *J = 7.98*, Ar-*H)*, 8.00 -8.15 (m, 2H, Ar-*H*), 9.49 (brs, 2H, NH_2,_ D_2_O exchangeable); ^13^C-NMR (125 MHz, DMSO-*d*_6_) δ ppm: 90.54 (CN), 115.65, 121.83, 128.33, 130,16, 133.84, 135.74, 141.12, 141.58, 144.78, 154.77, 157.98, 158.09, 161.35. Its MS (m/z), 307 (M^+^, 88%); C_17_H_10_FN_3_S (307.3); Anal. Calcd.; % C: 66.43, % H: 3.28, % N: 13.67; found % C: 66.40, % H: 3.26, % N: 13.66.

1-amino-2-(piperon-2-yl)pyrrolo[2,1-b][1,3]benzothiazole-3-carbonitrile (**9e**).

White powder, mp. 248–250 °C; IR (KBr, cm^−1^); 3429, 3409 (NH_2_), 3080 (CH aryl), 2985, 2868 (CH aliphatic), 2253(CN); ^1^H NMR (500 MHz, DMSO-*d*_6_, δ, ppm); 6.08 (s, 2H, O-CH_2_-O), 7.41–7.51 (m, 3H, Ar-*H*), 7.73 (s, 1H, Ar-*H*), 7.88–7.89 (m, 1H, Ar-*H*), 8.04–8.12 (m, 2H, Ar-*H*), 9.42 (brs, 2H, NH_2_, D_2_O exchangeable); ^13^C-NMR (125 MHz, DMSO-d_6_) δ ppm: 92.32 (CN), 102.99, 115.84, 128.36, 130.03, 133.57, 136.77, 158.67, 159.08, 159.44, 161.71, 162.00, 162.89. Its MS (m/z), 333 (M^+^, 92%); C_18_H_11_N_3_O_2_S (333.3); Anal. Calcd.; % C: 64.85, % H: 3.33, % N: 12.60; found % C: 64.83, % H: 3.30, % N: 12.58.

### 3.3. Biological Evaluation

#### 3.3.1. Antimicrobial Activity 

The antimicrobial activity of the series of newly synthesized compounds **5a**–**e**, **7a**–**e** and **9a**–**e** was evaluated in vitro against three Gram-positive bacteria Staphylococcus aureus ATCC-23444, Streptococcus pneumonia ATCC 22038 and Bacillus subtilis ATCC 24431, three Gram-negative bacteria Chlamydia pneumoniae ATCC-26452, Escherichia coli MTCC 23489, and Salmonella typhi ATCC 24351 as well as three fungi Aspergillus flavus (ATCC-24866, Candida albicans ATCC 24328 and Ganoderma lucidum ATCC-46816. All microorganisms were purchased from the American Type Culture Collection (Manassas, VA, USA). Using agar disk diffusion technique [41], using cefotaxime (109.8 μmol L^–1^) and fluconazole (15.313 μmol L^–1^) as reference drugs for antibacterial and antifungal activity, respectively. A solution of 100 µg mL^–1^ of the test compound was applied to microplate-wells, 1 cm in diameter. Inhibition zones were measured with calipers or automated scanners after 24 h incubation at 37 °C and compared with the standard. Minimum inhibitory concentration (*MIC* μmol L^–1^), of all synthesized compounds was carried out by using serial plate dilution technique [42]. Serial dilutions were prepared from the stock solution by dissolving 5 mg of each test compound in 1 mL of DMSO. The plates were incubated at 37 °C for 24 h. DMSO was used as a solvent control which had no effect on bacterial growth. Results of antimicrobial activities are summarized in Table 6 and Table 7.

#### 3.3.2. Antitumor Activity

Human carcinoma cell lines (lung cell NCI-H460, liver cancer HepG2 and colon cancer HCT-116) were provided by the National Cancer Institute (NCI, Cairo, Egypt). (FBS) fetal bovine serum from Gibco Invitrogen Co. (UK). Dimethyl sulfoxide (DMSO) and doxorubicin were from Sigma Chemical Co. (USA).

##### In Vitro Anticancer Activity by MTT Assay

Cell culture: All the synthesized compounds were tested in vitro for anticancer activity against three tumor cell lines (lung cell NCI-H460, liver cancer HepG2 and colon cancer HCT-116). Cells were cultured in humidified atmosphere at 37 °C with 5% CO_2_. The cell density was 2 × 10^3^ seeded in a 96-multiwell plate in 0.1mL DMEM (Dulbecco’s Modified Eagle’s Medium) supplemented with 10% (FBS) fetal bovine serum for 24 h. The tested compounds were dissolved in DMSO as a stock solution (0.1 mol L^–1^). After 48 h of incubation, cells were treated with different concentrations of the tested compounds (5, 12, 25, and 50 μmol L^−1^). The medium cells of the control group contained 0.2% DMSO. For each individual dose triplicate wells were performed. To each well, MTT in phosphate buffered saline (PBS, 5 mg/mL) was added and incubated for 4 h at 37 °C. After that, MTT reagent was removed and added (DMSO, 100 µL) to each well to dissolve formazan crystals. A measure of 100μM of Doxorubicin was used as a standard drug and the optical density was determined at 570 nm with a DTX 880 multimode detector. The calculation of the IC50 was done by performing 4 concentrations of the compounds on the cells in triplicate and the results were analyzed using SPSS software (Version 20.0).

#### 3.3.3. Antioxidant ABTS^•+^ Radical Activity

The ABTS**^•^**^+^ isolation activity was described by Dorman and Hiltunen [43]. A sample (2 mM) of ABTS (3-ethylbenzthiazoline-6-sulfonic acid) was treated with potassium persulfate (2.45 mM) to produce ABTS**^•^**^+^ solution and then incubated in dark room at room temperature for 16 h. The absorbance (*A*_0_) of the resulting green-blue solution (ABTS**^•^**^+^) was adjusted before use at 0.7 ± 0.05 nm at λ 734 nm. The solution of (50 µL of 2 mM) of the tested compounds in spectroscopic grade MeOH/phosphate buffer (1:1) was treated with ABTS**^•^**^+^. Afterwards, the absorbance (*A*_1_) was measured, then the % inhibition was calculated as

ABTS^•+^ scavenging effect % = [(A_0_ − A_1_)\A_1_] × 100

Trolox was used as standard antioxidant (positive control). The results (IC50 value) were calculated. 

#### 3.3.4. Bleomycin-Dependent DNA Damage

Assay mixtures [44] contained DNA (0.5 mg/mL), bleomycin sulfate (0.05 mg/mL), FeCl_3_ (50 mM), MgCl_2_ (5 mM), and selected synthesized compounds to be examined at different concentrations. To start the reaction, Trolox was added which was used as positive control and was incubated at 37 °C for 1 h. After the incubation period, 0.05 mL of EDTA (0.1 M) was added to terminate the reaction. the addition of 0.5 mL of (TBA) thiobarbituric acid (1%, *w/v*) and 0.5 mL HCI (25%, *v/v*) then heated in the water bath for 10 min at 80 °C to assay DNA damage. the extent of DNA damage was measured after centrifugation, by the increase in absorbance at 532 nm. 

## 4. Conclusions

The present investigation offers effective and rapid new procedures for the synthesis of the newly polycondensed heterocyclic ring systems. All the newly synthesized compounds showed antimicrobial activity against bacteria and fungi. Among the synthesized compounds, compounds **7a**,**d**, and **9a**,**d** showed more potent inhibitory activities than cefotaxime and fluconazole while the remaining compounds exhibited good to moderate activity against bacteria and fungi. Compounds **7a**,**d,** and **9a**,**d** also exhibited higher cytotoxicity for all tested cell lines compared to doxorubicin. These derivatives exhibited higher antioxidant activity compared to Trolox, also manifested the best protective effect against DNA damage induced by Bleomycin. Based on the anticancer and antioxidant activity, we can consider 2,3-dihydropyrido[2,3-d]pyrimidine-4-one and pyrrolo[2,1-b][1,3]benzothiazole as highly potential molecules for the development of novel anticancer drugs.

## Figures and Tables

**Table 1:** Microwave-assisted reaction of 2-cyanomethyl-1,3-benzothiazol and aldehydes.

Entry	Substrate	Product	Time	Yield (%)
**1**	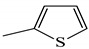	**5a**	6 min	87
**2**	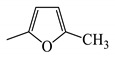	**5b**	6 min	84
**3**	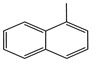	**5c**	8 min	90
**4**	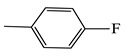	**5d**	4 min	96
**5**	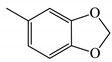	**5e**	8 min	92

**Table 2:** Reaction conditions: reaction of 2-(1,3-benzothiazol-2-yl)-3-(aryl)prop-2-enenitrile derivatives (**5a**–**e**) and 6-aminothiouracil **6** was heated or irradiated by microwave.

Entry	Substrate	Solvent	Method/Time		Method/Yield %		Product
			Conventional, h MWI, min		Conventional, h MWI, min		
**1**	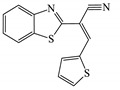	DMF	28	25	62	89	**7a**
		EtOH/TEA	36	45	48	53	
**2**	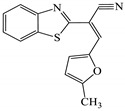	DMF	27	25	60	85	**7b**
		EtOH/TEA	36	45	43	51	
**3**	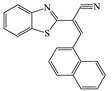	DMF	28	25	63	88	**7c**
		EtOH/TEA	40	45	41	49	
**4**	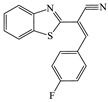	DMF	25	25	65	92	**7d**
		EtOH/TEA	35	45	48	53	
**5**	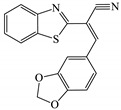	DMF	30	25	60	84	**7e**
		EtOH/TEA	42	45	39	50	

**Table 3:** Multicomponent reaction conditions by method of heating or microwave irradiation.

Comp.	Reagents			Method\Reaction Time		Method/Yield (%)	
	3	6	4	Conventional, h	MWI, min	Conventional	MWI
**7a**	**3**	**6**	**4a**	30	50	51	65
**7b**	**3**	**6**	**4b**	28	50	59	68
**7c**	**3**	**6**	**4c**	30	50	53	70
**7d**	**3**	**6**	**4d**	28	50	62	73
**7e**	**3**	**6**	**4e**	28	50	44	63

**Table 4:** One pot three-component reaction optimization for the synthesis of 1-amino-pyrrolobenzothiazole-3-carbonitrile derivatives **9a**–**e**.

Entry	3 eq.	8 eq.	4 eq.	Solvent	Method/(T °C)	Time, min	Yield (%)
**1**	1	1	4d, 1	MeOH	MV, 100 °C	25	61
**2**	1	1	4d, 1	DCE	MV, 100 °C	25	NR
**3**	1	1	4d, 1	EtOH	MV, 100 °C	25	68
**4**	1	1	4d, 1	EtOH/TEA	MV, 100 °C	25	73
**5**	1	1	4d, 1	EtOH/TEA	MV, 120 °C	25	78
**6**	1	1	4d, 1	EtOH/DBU(1 eq.)	MV, 100 °C	25	75
**7**	1	1	4d, 1	EtOH/DBU(1 eq.)	MV, 120 °C	25	87
**8**	1	1	4a, 1	EtOH/DBU(1 eq.)	MV, 120 °C	25	72
**9**	1	1	4b, 1	EtOH/DBU(1 eq.)	MV, 120 °C	25	70
**10**	1	1	4c, 1	EtOH/DBU(1 eq.)	MV, 120 °C	25	71
**11**	1	1	4e, 1	EtOH/DBU(1 eq.)	MV, 120 °C	25	66
**12**	1	1	4a, 1	EtOH/TEA	Heating	20 h	62
**13**	1	1	4b, 1	EtOH/TEA	Heating	22 h	59
**14**	1	1	4c, 1	EtOH/TEA	Heating	20 h	63
**15**	1	1	4d, 1	EtOH/TEA	Heating	19 h	68
**16**	1	1	4e, 1	EtOH/TEA	Heating	24 h	52

**Table 5:** Reaction condition for the synthesis of **9a**–**e**.

Comp.	Reagents		Method\Reaction Time		Method/Yield (%)	
	5	8	Conventional, h	MWI, min.	Conventional	MWI
**9a**	**5a**	**8**	15	25	70	82
**9b**	**5b**	**8**	13	20	68	79
**9c**	**5c**	**8**	15	22	70	85
**9d**	**5d**	**8**	12	21	74	90
**9e**	**5e**	**8**	14	23	65	78

**Table 6:** Minimum inhibitory concentration (*MIC*, μmol L^–1^) of newly synthesized compounds against bacteria.

Entry	Gram-Positive Bacteria			Gram-Negative Bacteria		
	*Staphylococcus Aureus*	*Streptococcus Pneumonia*	*Bacillus Subtilis*	*Chlamydia Pneumonia*	*Escherichia Coli*	*Salmonella Typhi*
**5a**	14	10	10	18	12	12
**5b**	16	16	19	16	15	20
**5c**	19	16	12	19	18	15
**5d**	12	9	8	16	13	15
**5e**	20	18	18	17	14	12
**7a**	8	4	6	12	5	10
**7b**	12	10	8	15	12	14
**7c**	12	10	10	14	10	10
**7d**	6	5	6	10	4	8
**7e**	14	12	9	12	10	10
**9a**	8	10	6	12	8	10
**9b**	14	10	10	14	10	12
**9c**	16	12	12	14	10	10
**9d**	10	4	6	8	5	8
**9e**	14	10	10	13	10	12
**Cefotaxime**	10	6	6	12	6	8

**Table 7:** Minimum inhibitory concentration (*MIC*, μmol L^–1^) of newly synthesized compounds against fungi.

Comp.	*Aspergillus Flavus*	*Candida Albicans*	*Ganoderma Lucidum*
**5a**	10	12	10
**5b**	11	13	14
**5c**	14	18	10
**5d**	12	12	13
**5e**	9	10	12
**7a**	8	10	9
**7b**	12	15	18
**7c**	12	16	12
**7d**	8	10	10
**7e**	9	18	20
**9a**	8	4	8
**9b**	9	10	12
**9c**	16	20	11
**9d**	6	5	4
**9e**	10	10	10
**Fluconazole**	6	8	6

**Table 8:** Inhibition of the growth of human lung cell NCI-H460, liver cancer (HepG2), colon cancer HCT-116 tumor cell lines by synthesized compounds.

Comp.	IC_50_ (μmol L^−1^) ^a^		
	NCI-H460	HepG2	HCT-116
**5a**	3.61 ± 0.28	3.14 ± 0.16	4.20 ± 0.75
**5b**	5.90 ± 0.14	5.82 ± 0.16	6.42 ± 0.17
**5c**	10.20 ± 2.68	9.10 ± 2.26	9.54 ± 1.28
**5d**	3.04 ± 0.15	3.20 ± 0.18	3.38 ± 0.27
**5e**	7.50 ± 0.78	8.20 ± 0.44	9.30 ± 2.46
**7a**	0.66 ± 0.08	0.45 ± 0.04	0.70 ± 0.14
**7b**	2.56 ± 0.18	2.89 ± 0.19	2.68 ± 0.12
**7c**	4.09 ± 0.33	5.35 ± 0.60	5.58 ± 0.58
**7d**	0.28 ± 0.04	0.39 ± 0.04	0.14 ± 0.04
**7e**	2.60 ± 0.24	3.00 ± 0.13	3.08 ± 0.64
**9a**	1.10 ± 0.11	0.49 ± 0.06	0.89 ± 0.06
**9b**	3.20 ± 0.18	3.54 ± 0.62	3.89 ± 0.73
**9c**	6.08 ± 0.42	6.90 ± 0.13	7.20 ± 0.46
**9d**	0.45± 0.06	0.47 ± 0.06	0.59 ± 0.10
**9e**	3.90 ± 0.17	4.08 ± 0.63	4.50 ± 0.54
**Doxorubicin**	1.98 ± 0.17	0.52 ± 0.06	1.12 ± 0.13

^a^ IC_50_: 50% inhibitory concentration. Mean ± SEM of three independent experiments performed in triplicate.

**Table 9:** Antioxidant activity and bleomycin-dependent DNA damage assay.

Comp.	Absorbance	*ABTS Inhibition*(%)	*Bleomycin-Dependent DNA Damage*
**7a**	0.06	91.2	0.053
**7b**	0.20	78.3	0.121
**7c**	0.28	75.3	0.128
**7d**	0.03	92.8	0.045
**7e**	0.28	68.0	0.146
**9a**	0.10	88.7	0.069
**9b**	0.32	69.3	0.186
**9c**	0.22	65.5	0.179
**9d**	0.08	90.4	0.064
**9e**	0.26	72.2	0.132
**Trolox**	0.04	89.5	0.067

All experiments were performed three times.

## Data Availability

The data presented in this study are available.

## Supplementary Materials

The following are available online. Figure S1. ^1^H-NMR and ^13^C-NMR spectra for all compounds.

## Sample Availability

Samples of the compounds are not available from authors.
