# Supplementary material for: Synthesis and Antimicrobial, Anticancer and Anti-Oxidant Activities of Novel 2,3-Dihydropyrido[2,3-d]pyrimidine-4-one and Pyrrolo[2,1-b][1,3]benzothiazole Derivatives via Microwave-Assisted Synthesis
Source: Molecules. 2022 Feb 12;27(4):1246. doi: 10.3390/molecules27041246 (PMC8880104; doi:10.3390/molecules27041246)

Supporting Information for:

Design, Synthesis and antimicrobial, anticancer and Anti- oxidant activities of novel pyrido[2,3-d]pyrimidine and pyrrolobenzothiazole derivatives via Microwave-assisted synthesis

Aamal A. Al-Mutairi, Hend N. Hafez, Abdel-Rhman B. A. El-Gazzar and Marwa Y. A. Mohamed

1 Chemistry Department, Faculty of Science, Kingdom of Saudi Arabia; Imam Mohammad Ibn Saud Islamic University (IMSIU), P.O. Box 90950 Riyadh 11623, Kingdom of Saudi Arabia, aamutairi@imamu.edu.sa (AAA)

2 Photochemistry Department, (Heterocyclic & Nucleosides Unit), National Research Centre, 12622 Dokki, Cairo, dr.hendhafez@yahoo.com (HNH.) ; profelgazzar@yahoo.com (ABAG.)

3 Biology Department, Faculty of Science, Kingdom of Saudi Arabia; Imam Mohammad Ibn Saud Islamic University (IMSIU), P.O. Box 90950 Riyadh 11623, Kingdom of Saudi Arabia, Yousry_marwa@yahoo.com (MYAM)

1. ^{1}H NMR spectra of (2Z)-2-(1,3- benzothiazol-2-yl)-3-(thiophen-2-yl)prop-2-enenitrile (**5a**) **S3**

2. ^{1}H NMR spectra of (2Z)-2-(1,3- benzothiazol-2-yl)-3-(5-methylfuran-2-yl) prop-2-enenitrile (**5b**) **S4**
3. ^{1}H NMR spectra of (2Z)-2-(1,3- benzothiazol-2-yl)-3-(naphthalen-1-yl) prop-2-enenitrile (**5c**) **S5**
4. ^{1}H NMR spectra of (2Z)-2-(1,3- benzothiazol-2-yl)-3-(4-fluorophenyl) prop-2-enenitrile (**5d**) **S6**
5. ^{1}H NMR spectra of (2Z)-2-(1,3- benzothiazol -2-yl)-3- (piperon-2-yl) prop-2-enenitrile (**5e**) **S7**
6. ^{1}H NMR and ^{13}C-NMR spectra of 7-amino-6-(1,3-benzothiazol-2-yl)-5-(thiophen-2-yl)-2-thioxo-2,3-dihydropyrido[2,3-d]pyrimidin-4(1H)-one (**7a**) **S8**
7. ^{1}H NMR and ^{13}C-NMR spectra of 7-amino-6-(1,3-benzothiazol-2-yl)-5-(5-methyl-furan-2-yl)-2-thioxo-2,3-dihydro pyrido [2,3-d] pyrimidin-4(1H)-one (**7b**) **S9**
8. ^{1}H NMR and ^{13}C-NMR spectra of 7-amino-6-(1,3-benzothiazol-2-yl)-5-(naphthalen-1-yl)- 2-thioxo-2,3-dihydro-pyrido [2,3-d] pyrimidin-4(1H)-one (**7c**) **S10-S11**

9. ^{1}H NMR and ^{13}C-NMR spectra of 7-amino-6-(1,3-benzothiazol-2-yl)-5-(4-fluoro

-phenyl)-2-thioxo-2,3-dihydro-pyrido [2,3-d] pyrimidin-4(1H)-one (**7d**) **S12**

10. ^{1}H NMR and ^{13}C-NMR spectra of 7-amino-5-(piperon-2-yl)-6-(1,3-benzothia-zol-2-yl)- 2-thioxo-2,3-dihydropyrido [2,3-d] pyrimidin-4(1H)-one (**7e**) **S13**
11. ^{1}H NMR and ^{13}C-NMR spectra of 1-amino-2-(thiophen-2-yl) pyrrolo[2,1-b][1,3] benzothiazole-3-carbonitrile (**9a**) **S14**
12. ^{1}H NMR and ^{13}C-NMR spectra of 1-amino-2-(5-methylfuran-2-yl) pyrrolo[2,1-b][1,3]benzothiazole -3-carbonitrile (**9b**) **S15**
13. ^{1}H NMR and ^{13}C-NMR spectra of 1-amino-2-(naphthalen-1-yl) pyrrolo[2,1-b][1,3]benzothiazole-3-carbonitrile (**9c**). **S16 – S17**
14. ^{1}H NMR and ^{13}C-NMR spectra of 1-amino-2-(4-fluorophenyl) pyrrolo[2,1-b][1,3]benzothiazole-3-carbonitrile (**9d**) **S18**
15. ^{1}H NMR and ^{13}C-NMR spectra of 1-amino-2-(piperon-2-yl) pyrrolo[2,1-b][1,3]benzothiazole-3-carbonitrile (**9e**) **S19**

^{1}H NMR spectra of (2Z)-2-(1,3- benzothiazol-2-yl)-3-(thiophen-2-yl)prop-2-enenitrile (**5a**)

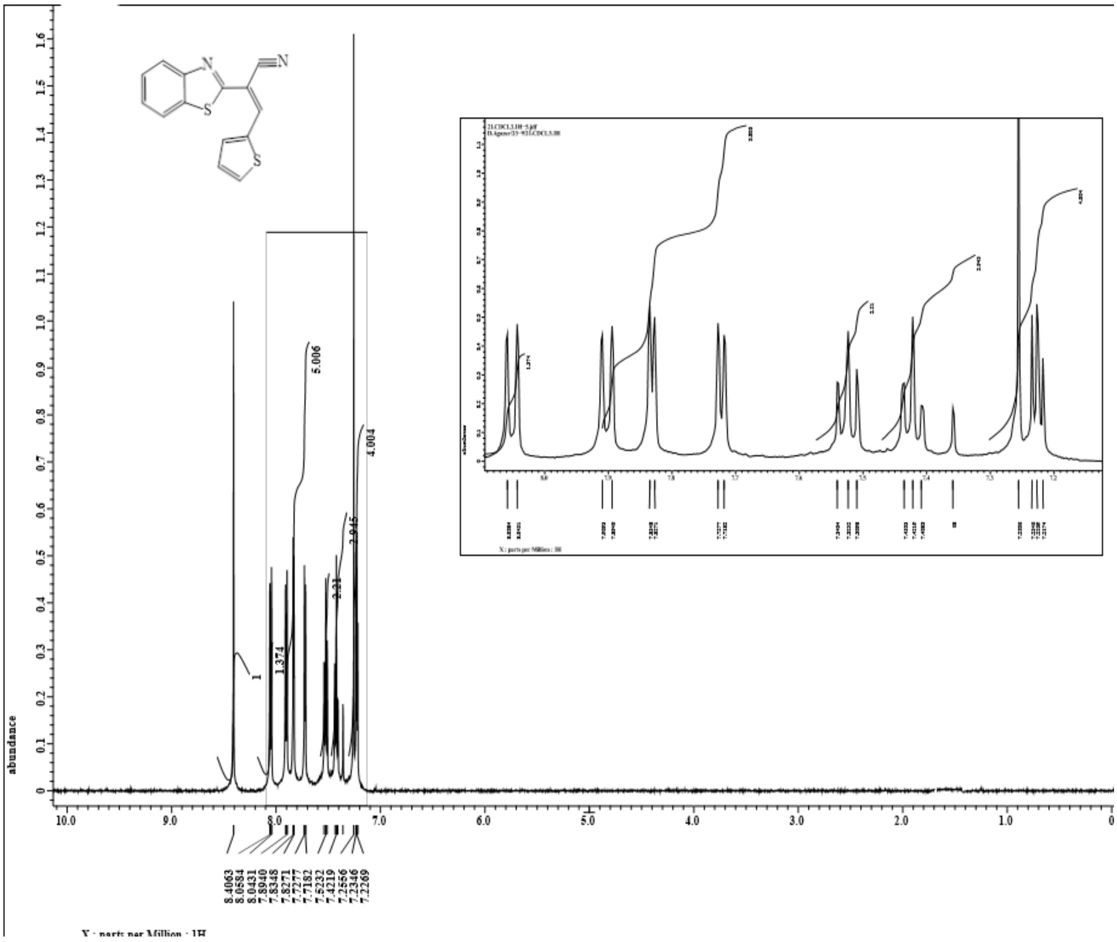

^{1}H NMR spectra of (2Z)-2-(1,3- benzothiazol-2-yl)-3-(5-methylfuran-2-yl) prop-2-enenitrile (**5b**)

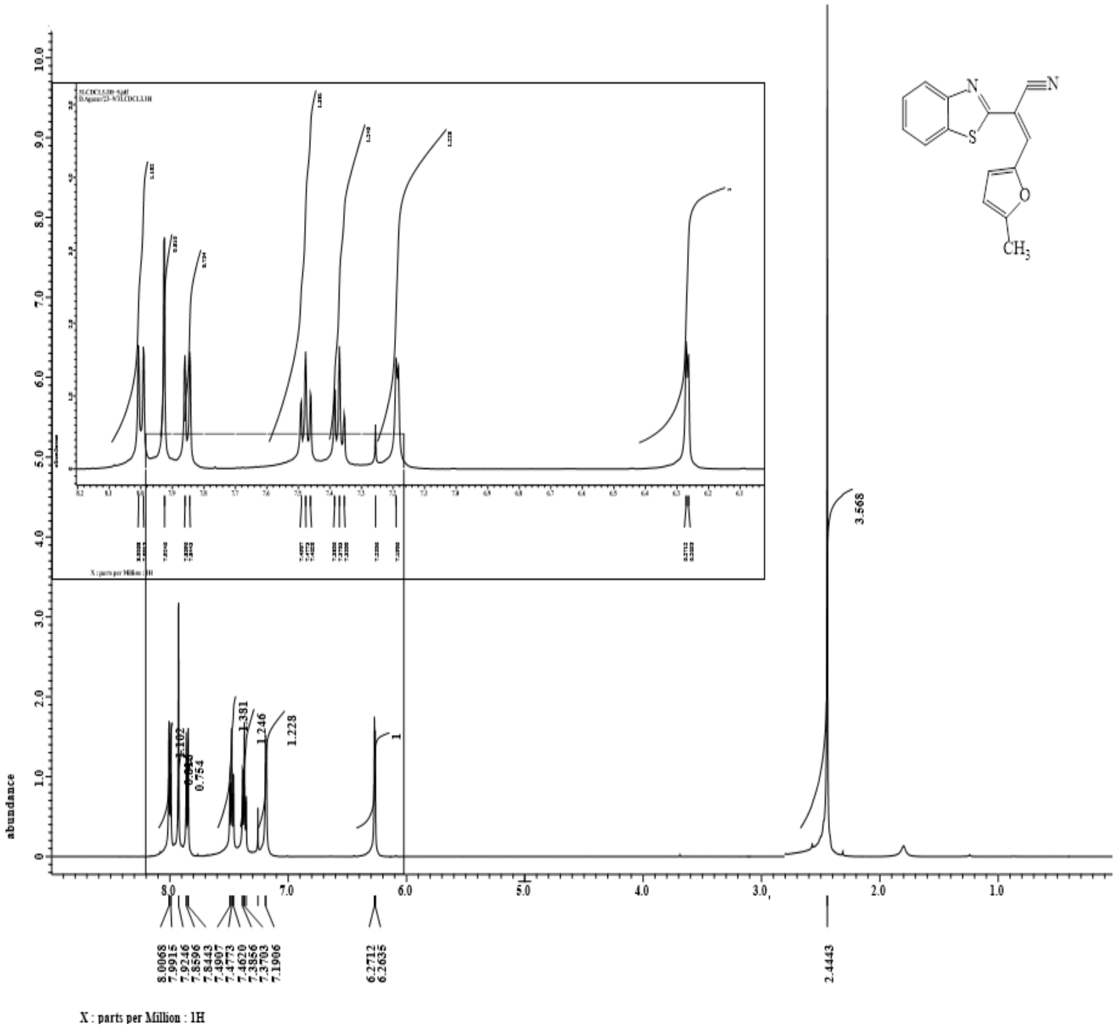

^{1}H NMR spectra of (2Z)-2-(1,3- benzothiazol-2-yl)-3-(naphthalen-1-yl) prop-2-enenitrile (**5c**)

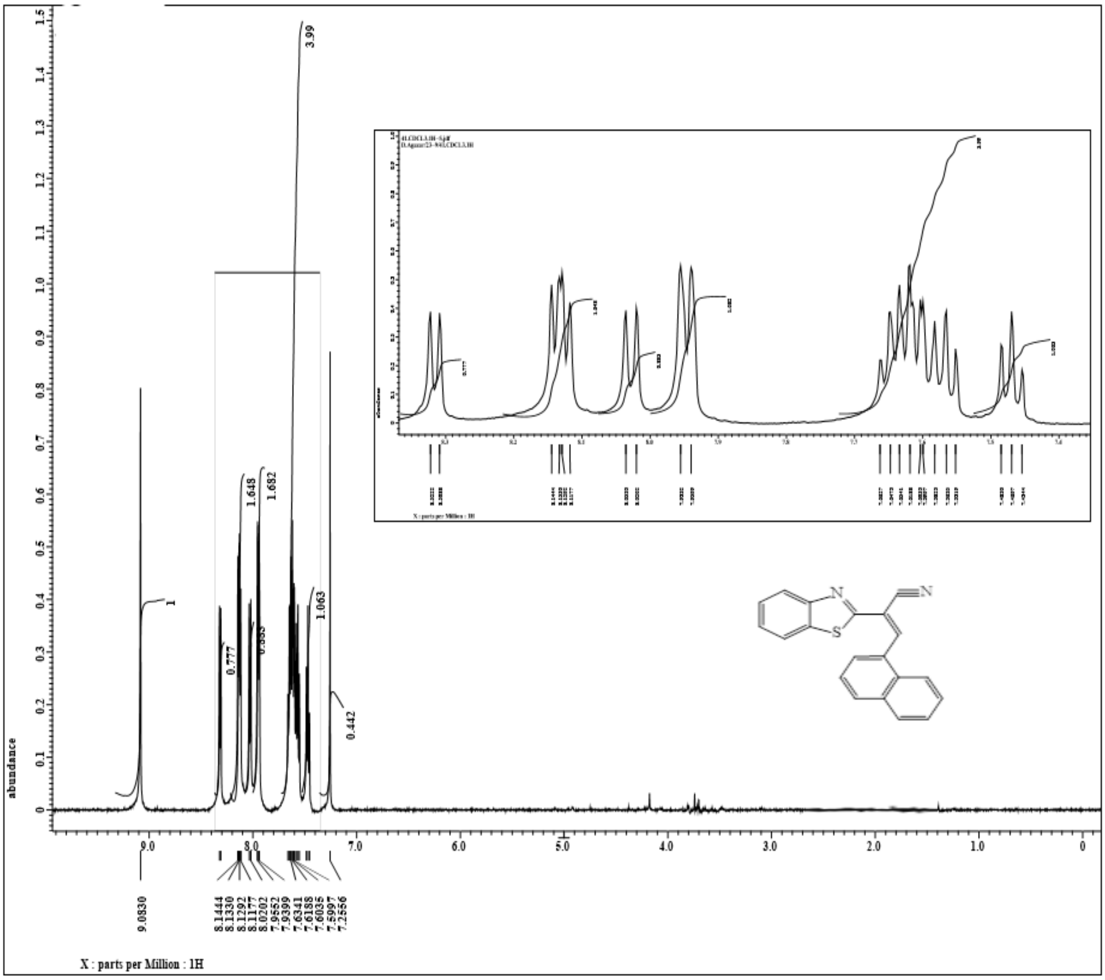

^{1}H NMR spectra of (2Z)-2-(1,3- benzothiazol-2-yl)-3-(4-fluorophenyl) prop-2-enenitrile (**5d**)

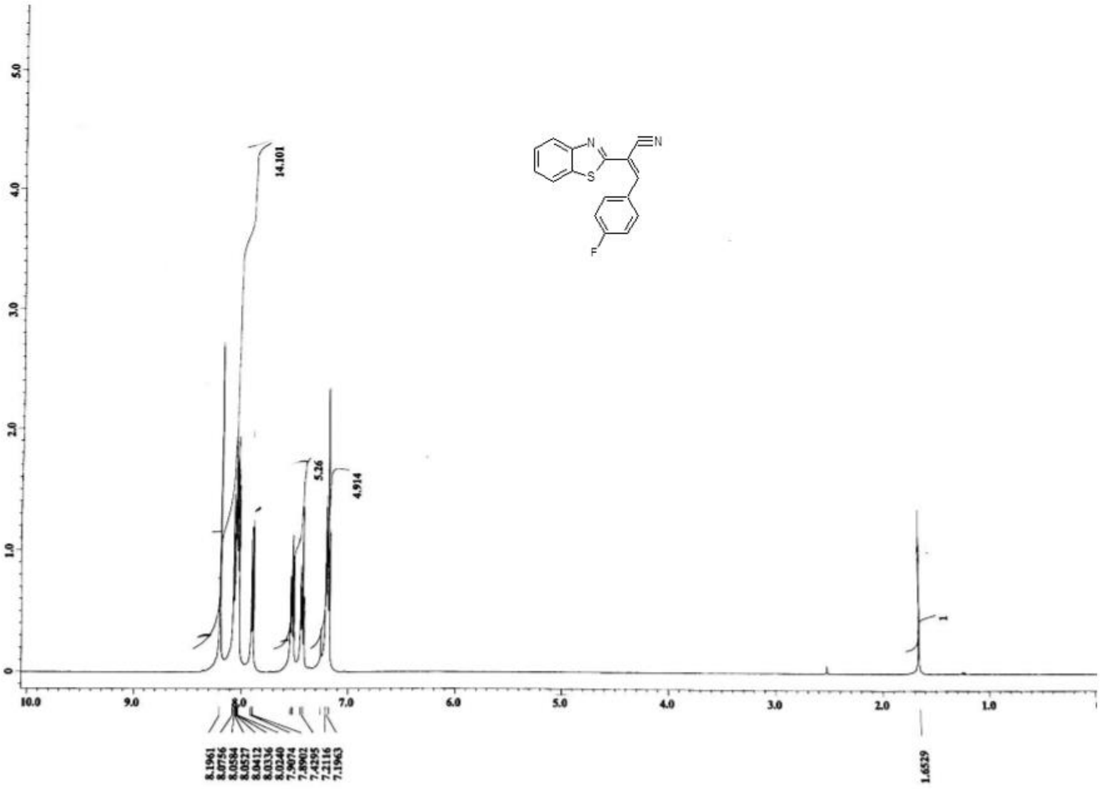

^{1}H NMR spectra of (2Z)-2-(1,3- benzothiazol -2-yl)-3- (piperon-2-yl) prop-2-enenitrile (**5e**)

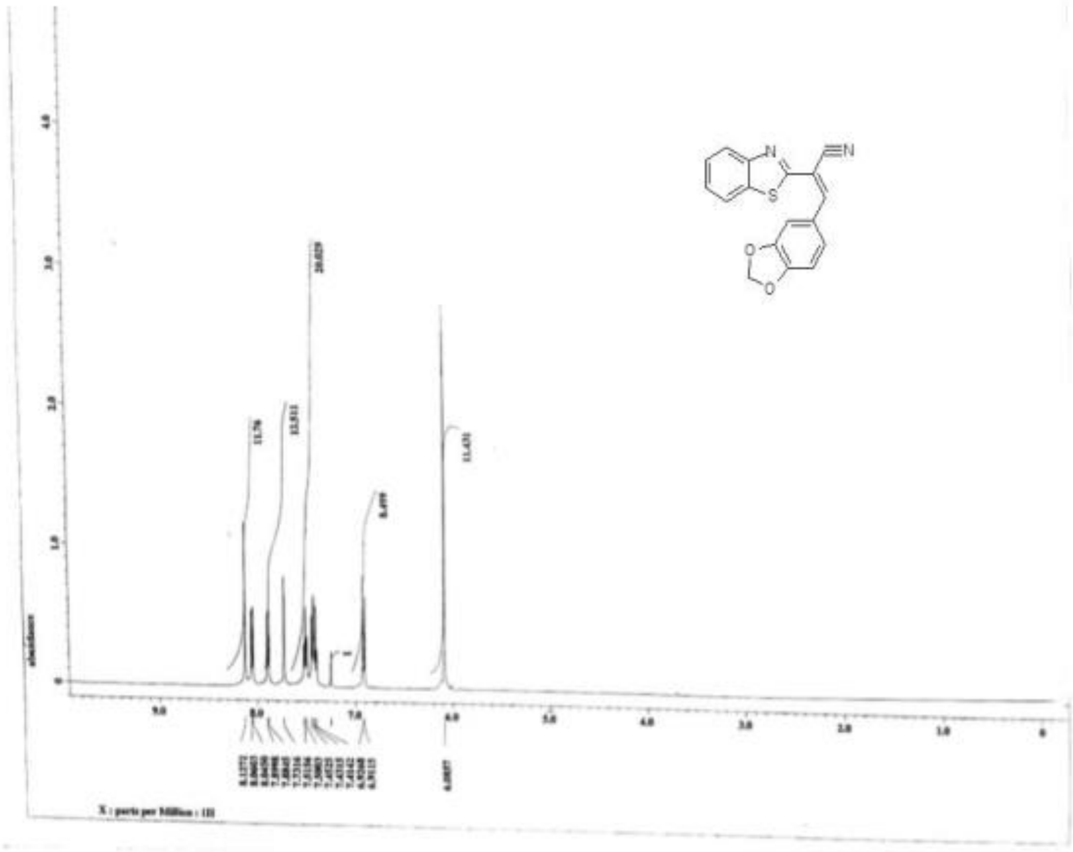

^{1}H NMR and ^{13}C-NMR spectra of 7-amino-6-(1,3-benzothiazol-2-yl)-5-(thiophen-2-yl)-2-thioxo-2,3-dihydropyrido[2,3-d]pyrimidin-4(1H)-one (**7a**)

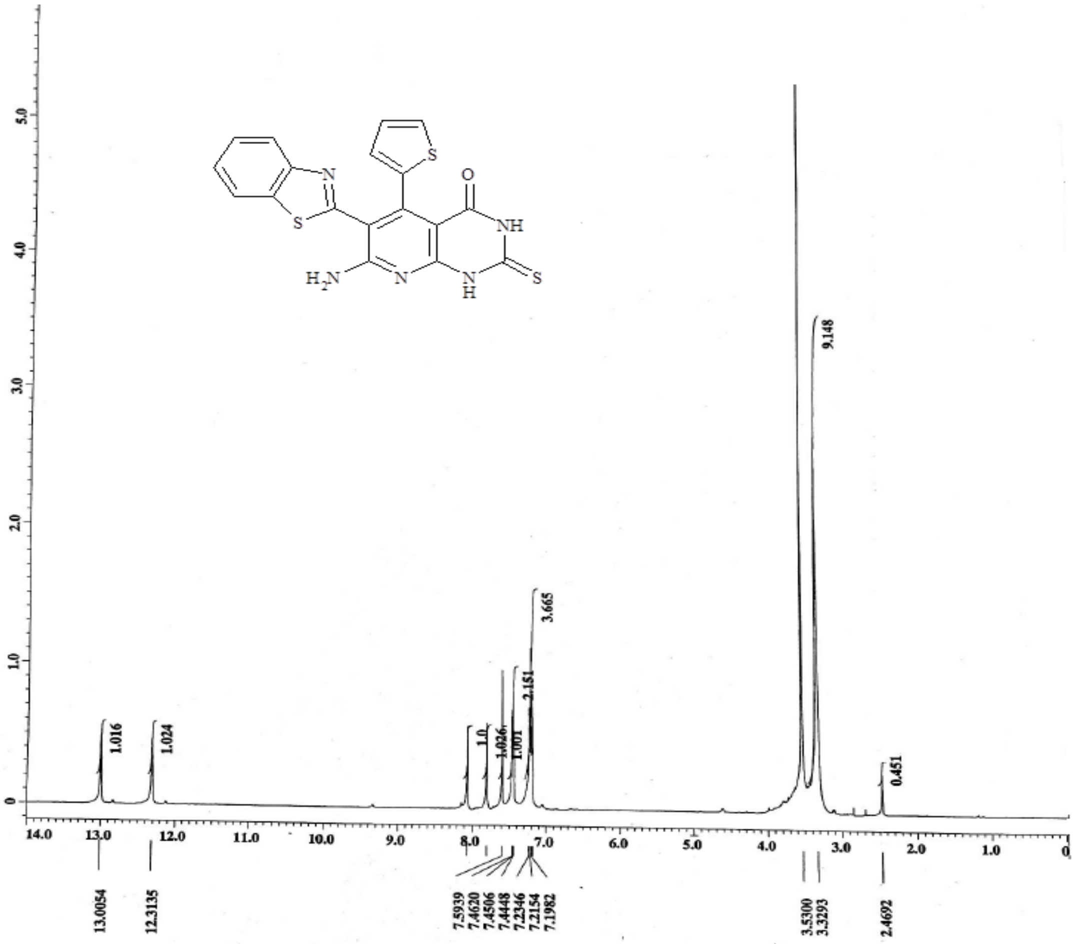

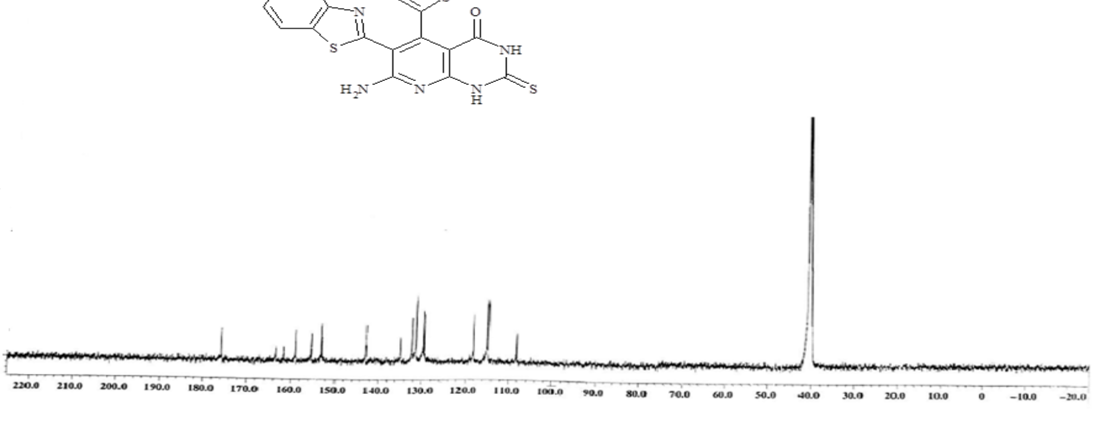

^{1}H NMR and ^{13}C-NMR spectra of 7-amino-6-(1,3-benzothiazol-2-yl)-5-(5-methyl-furan-2-yl)-2-thioxo-2,3-dihydro pyrido [2,3-d] pyrimidin-4(1H)-one **(7b)**

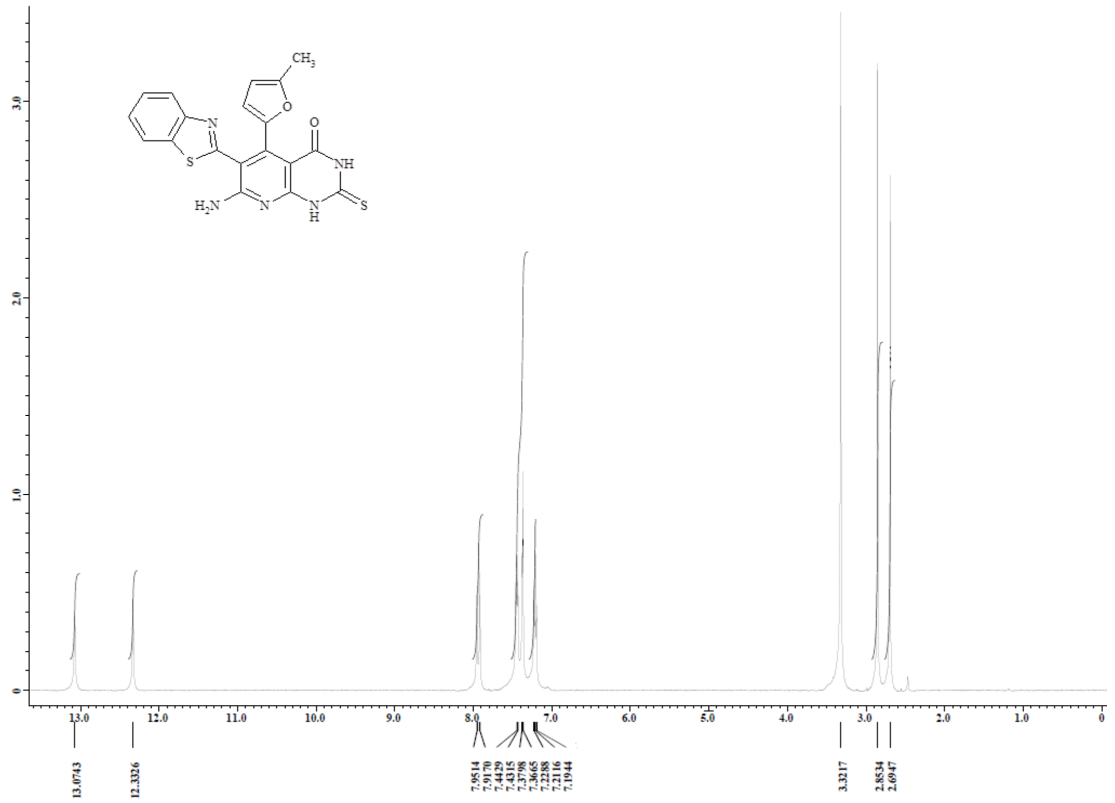

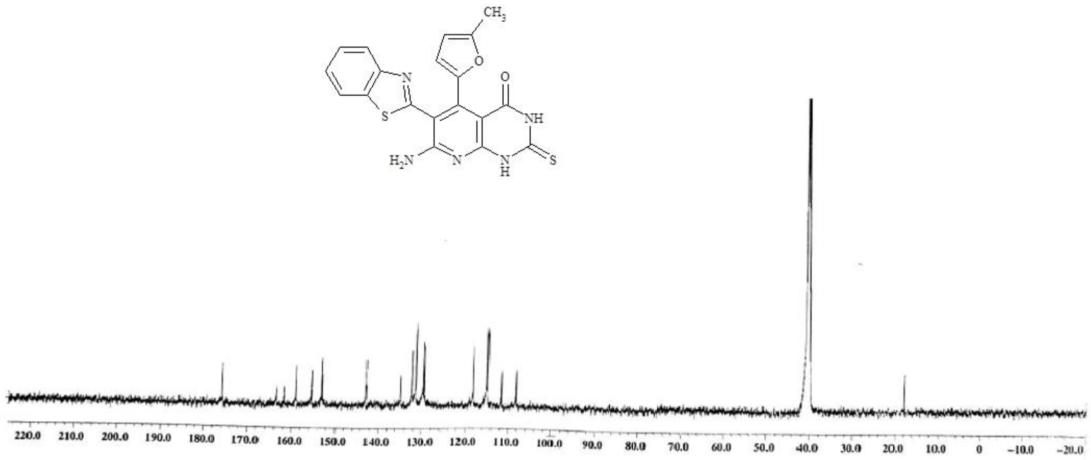

^{1}H NMR and ^{13}C-NMR spectra of 7-amino-6-(1,3-benzothiazol-2-yl)-5- (naphthalen – 1-yl)- 2-thioxo-2,3-dihydro-pyrido [2,3-d] pyrimidin-4(1H)-one (**7c**)

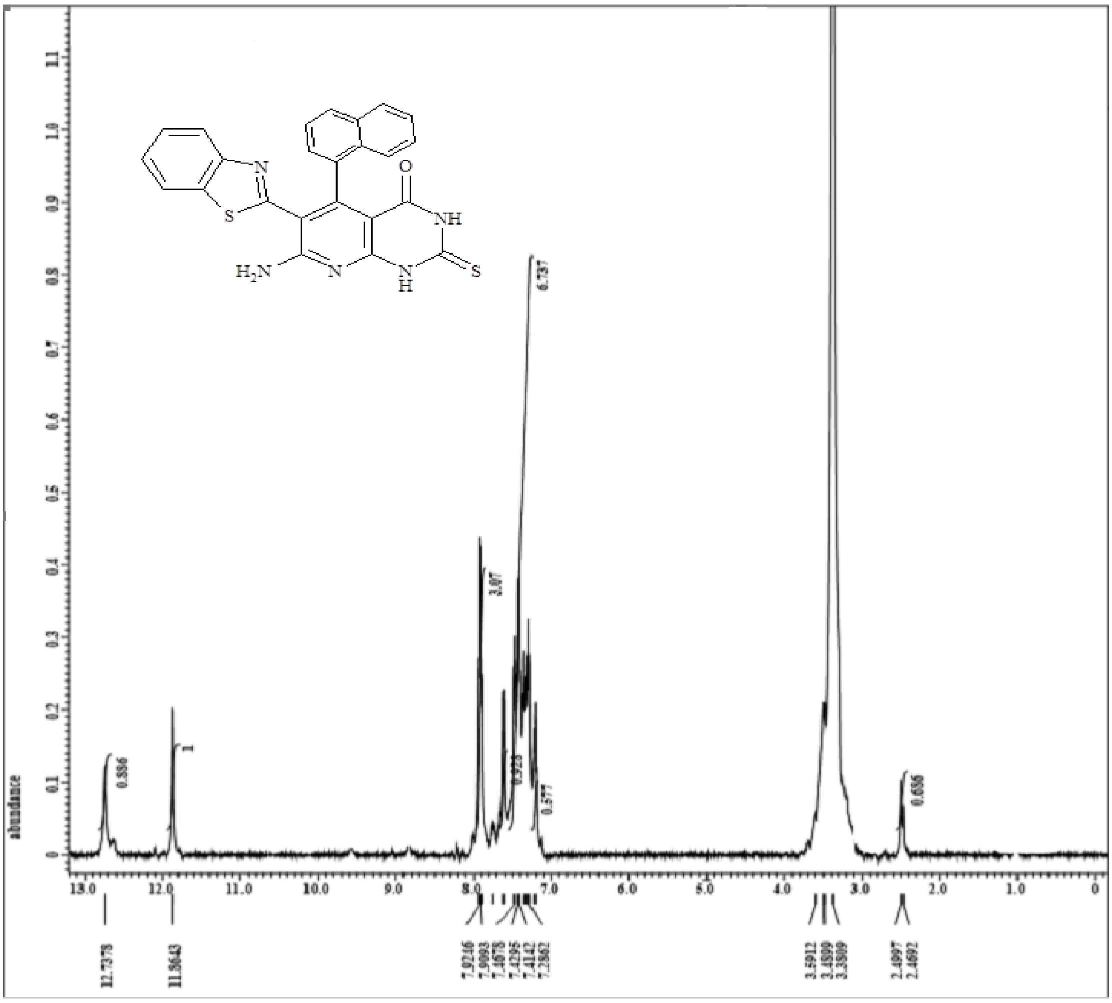

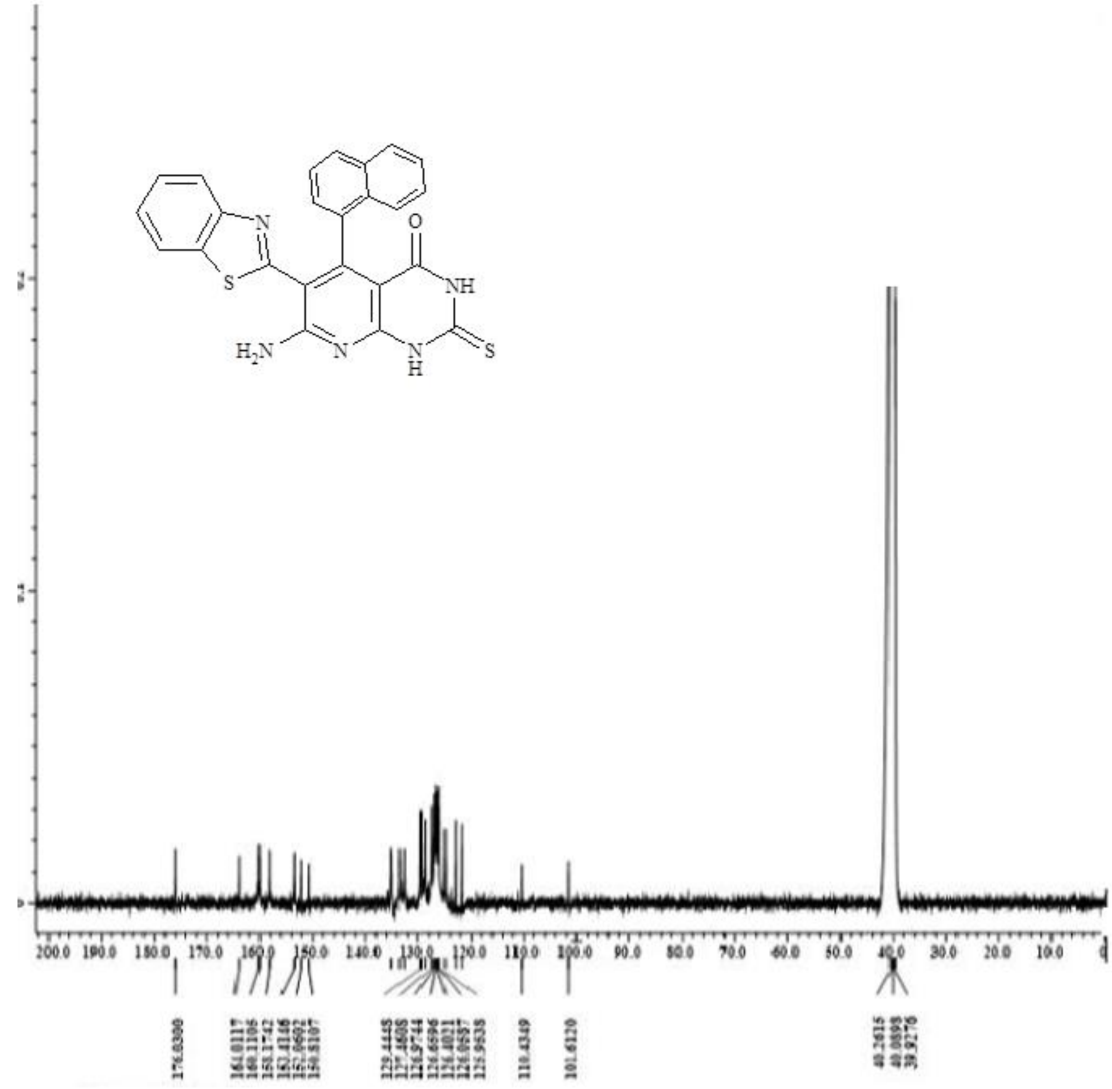

O
NH
S
H$_2$N
N
N
H
S
200.0 190.0 180.0 170.0 160.0 150.0 140.0 130.0 120.0 110.0 100.0 90.0 80.0 70.0 60.0 50.0 40.0 30.0 20.0 10.0 0
176.0300
164.0117
160.1106
158.1742
152.4146
151.0602
150.8107
129.4445
127.4605
126.9744
126.6596
126.4021
126.0957
125.9538
110.4349
101.6120
40.2615
40.0895
39.9276

^{1}H NMR and ^{13}C-NMR spectra of 7-amino-6-(1,3-benzothiazol-2-yl)-5-(4-fluoro -phenyl)-2-thioxo-2,3-dihydro-pyrido [2,3-d] pyrimidin-4(1H)-one (**7d**)

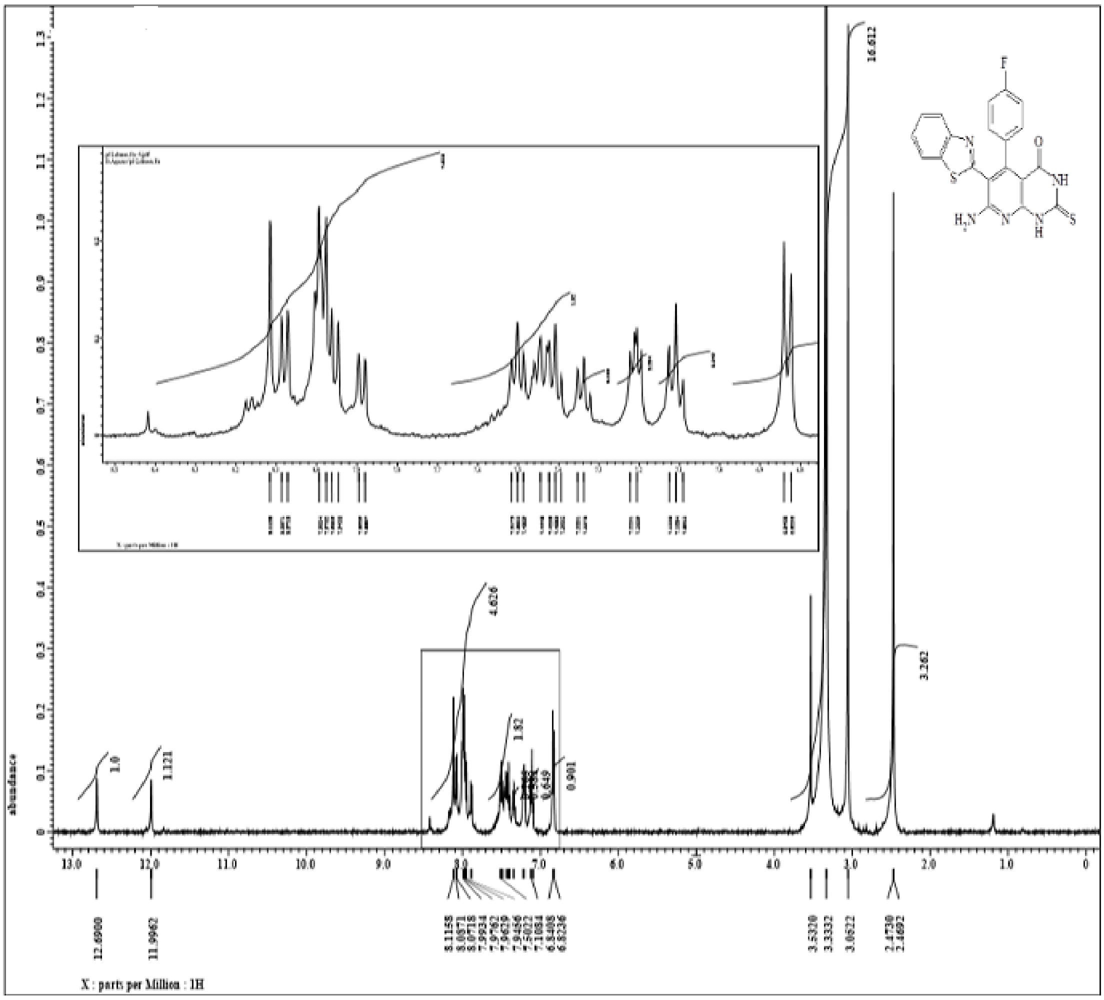

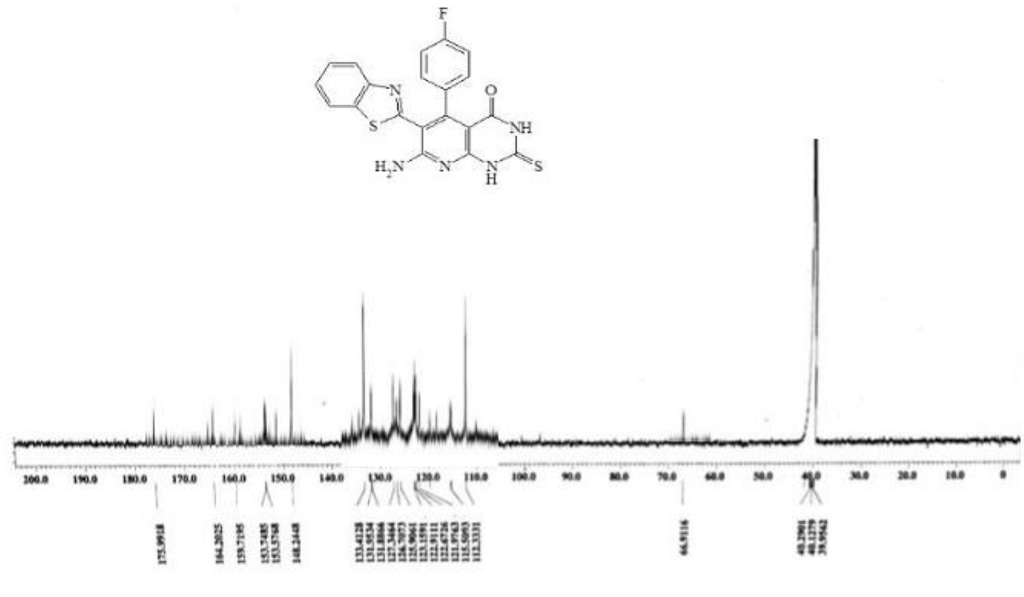

^{1}H NMR and ^{13}C-NMR spectra of 7-amino-5-(piperon-2-yl)-6-(1,3-benzothia-zol-2-yl)- 2-thioxo-2,3-dihydropyrido [2,3-d] pyrimidin-4(1H)-one (**7e**)

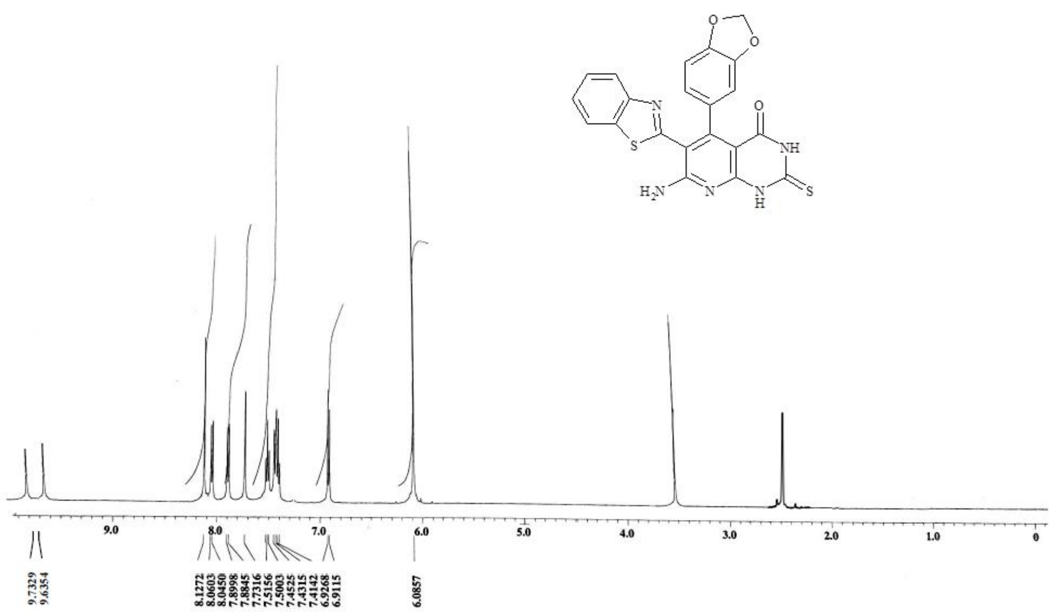

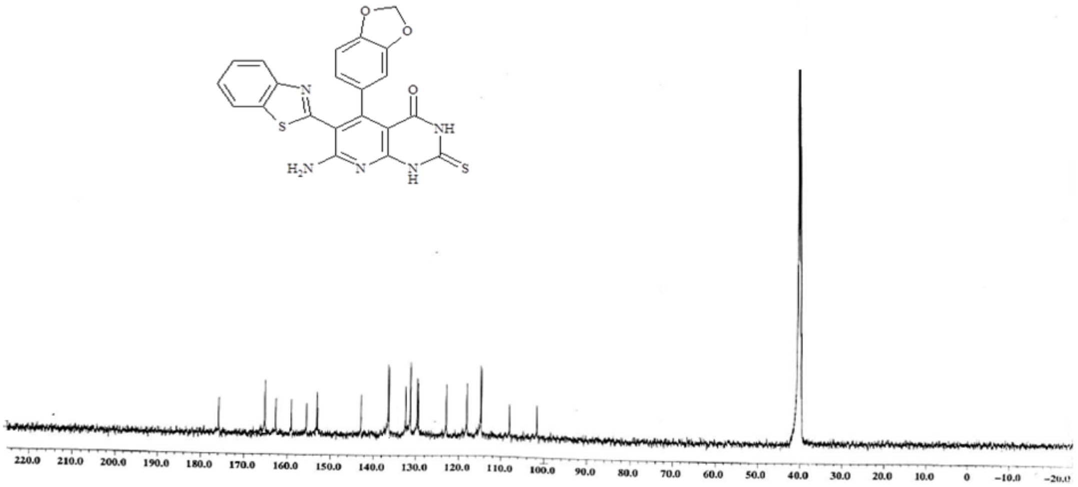

^{1}H NMR and ^{13}C-NMR spectra of 1-amino-2-(thiophen-2-yl) pyrrolo[2,1-b][1,3] benzothiazole-3-carbonitrile (**9a**)

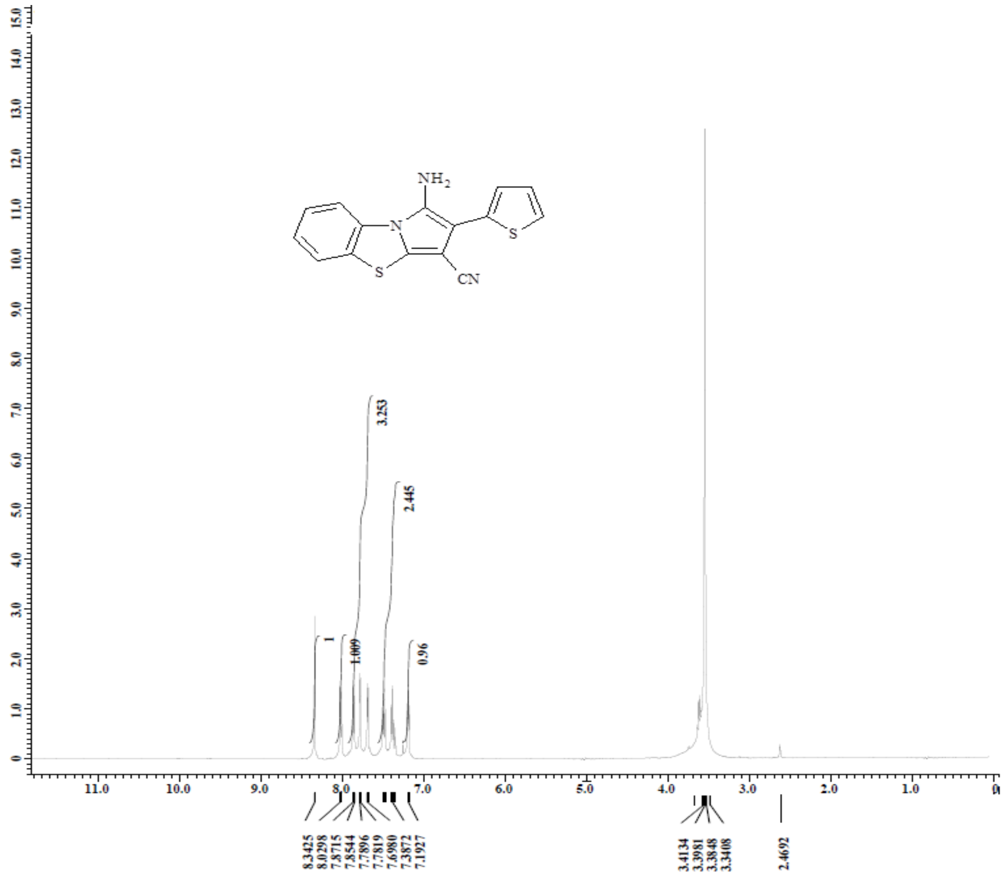

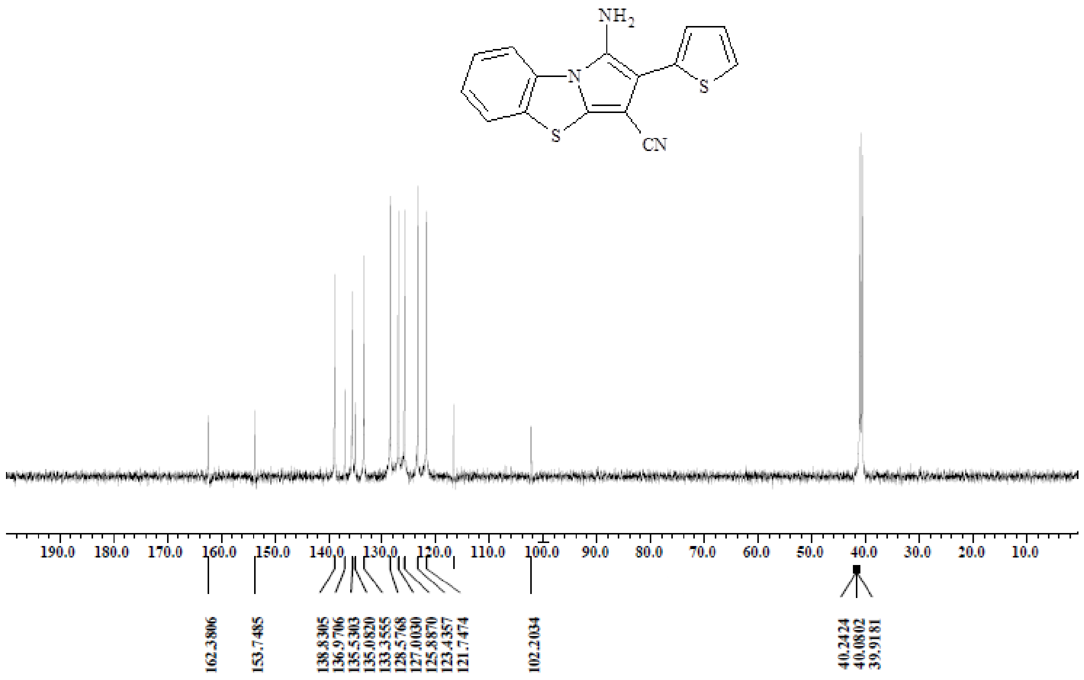

^{1}H NMR and ^{13}C-NMR spectra of 1-amino-2-(5-methylfuran-2-yl) pyrrolo[2,1-b][1,3]benzothiazole -3-carbonitrile (**9b**)

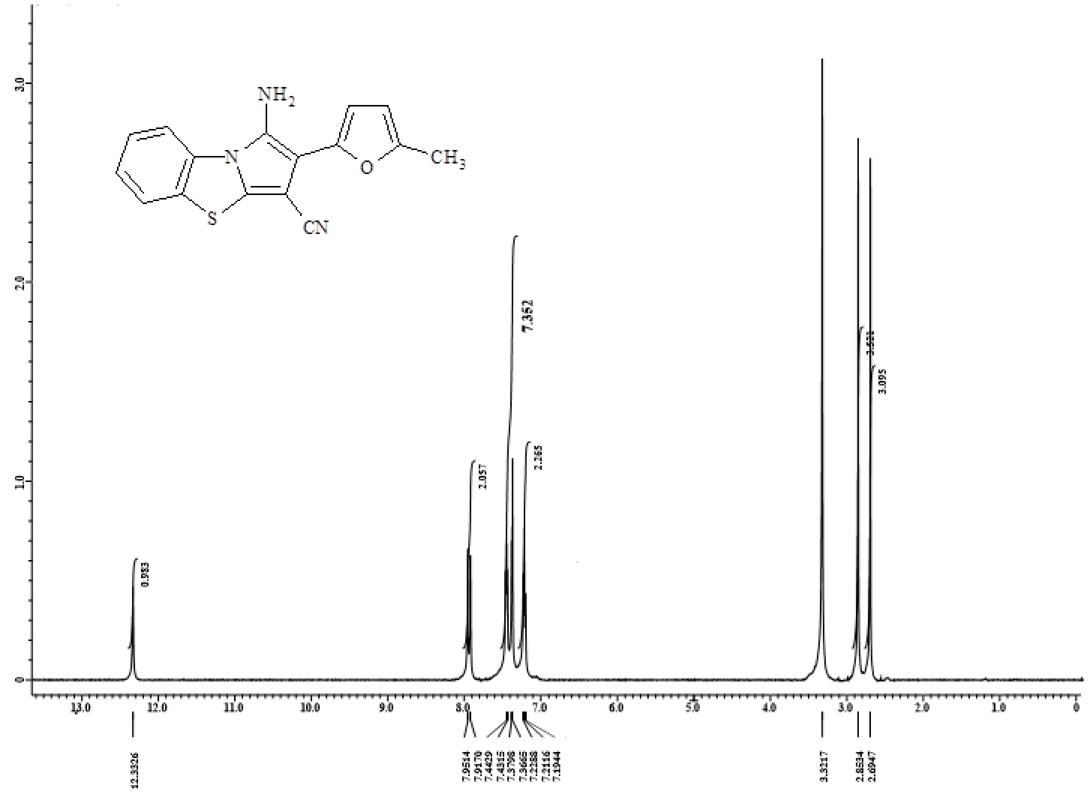

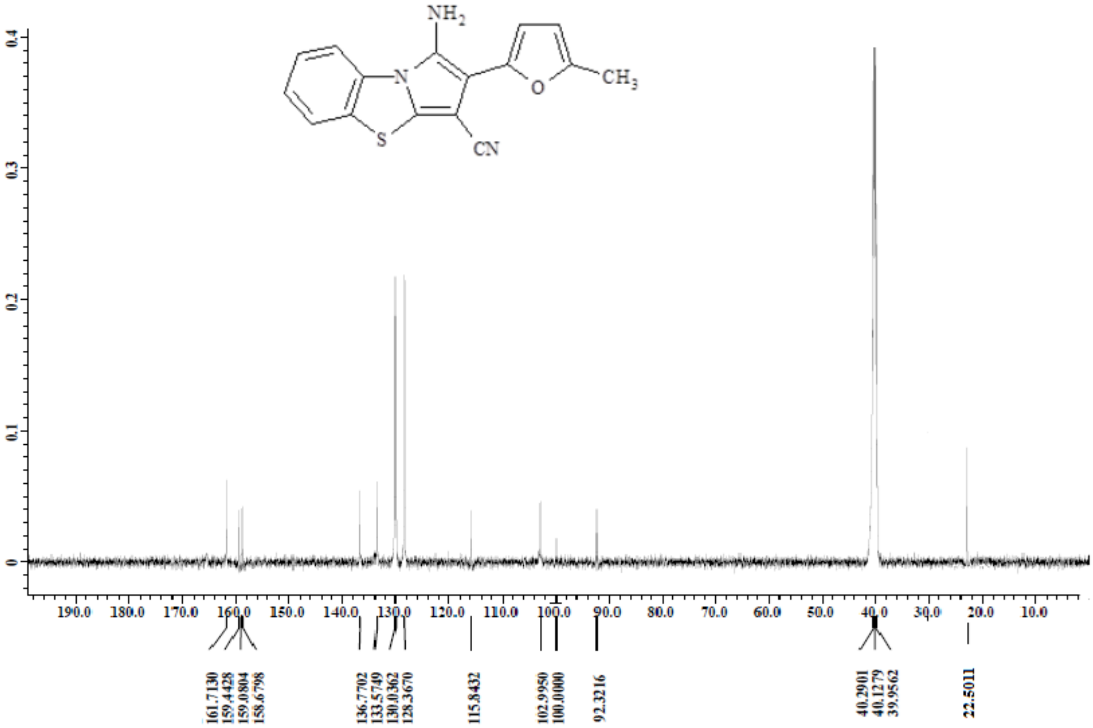

^{1}H NMR and ^{13}C-NMR spectra of 1-amino-2-(naphthalen-1-yl) pyrrolo[2,1-b][1,3] benzothiazole-3-carbonitrile (**9c**).

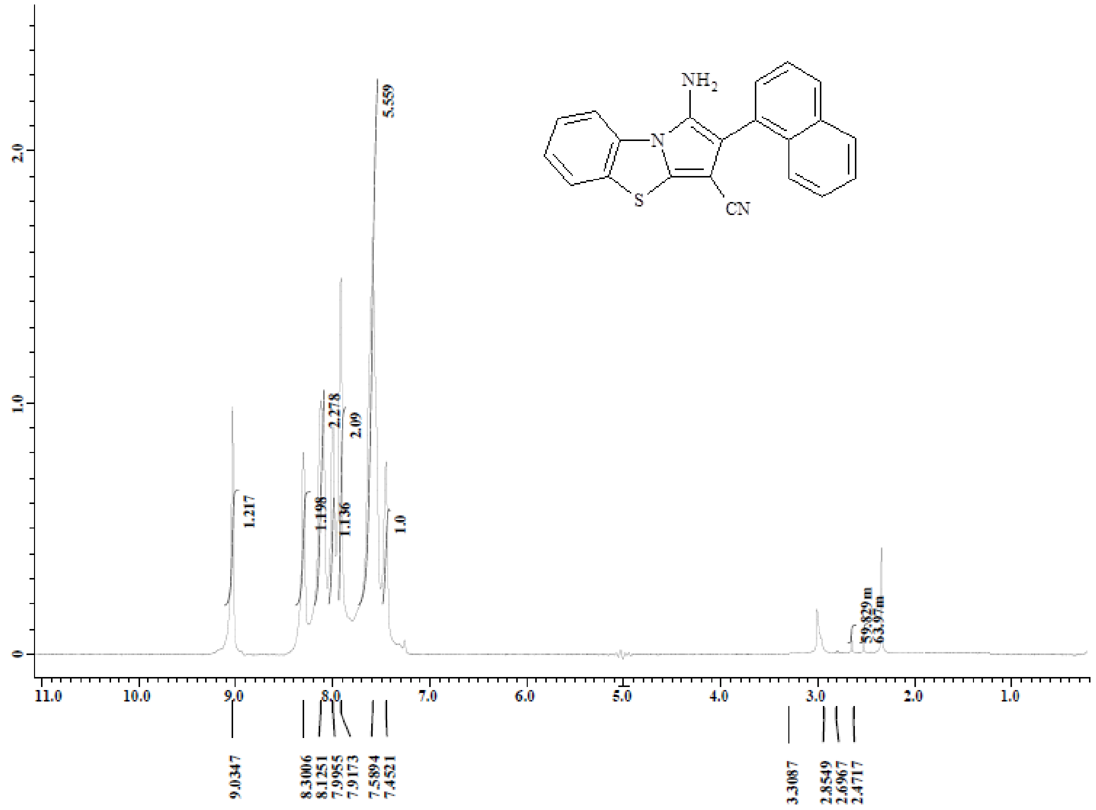

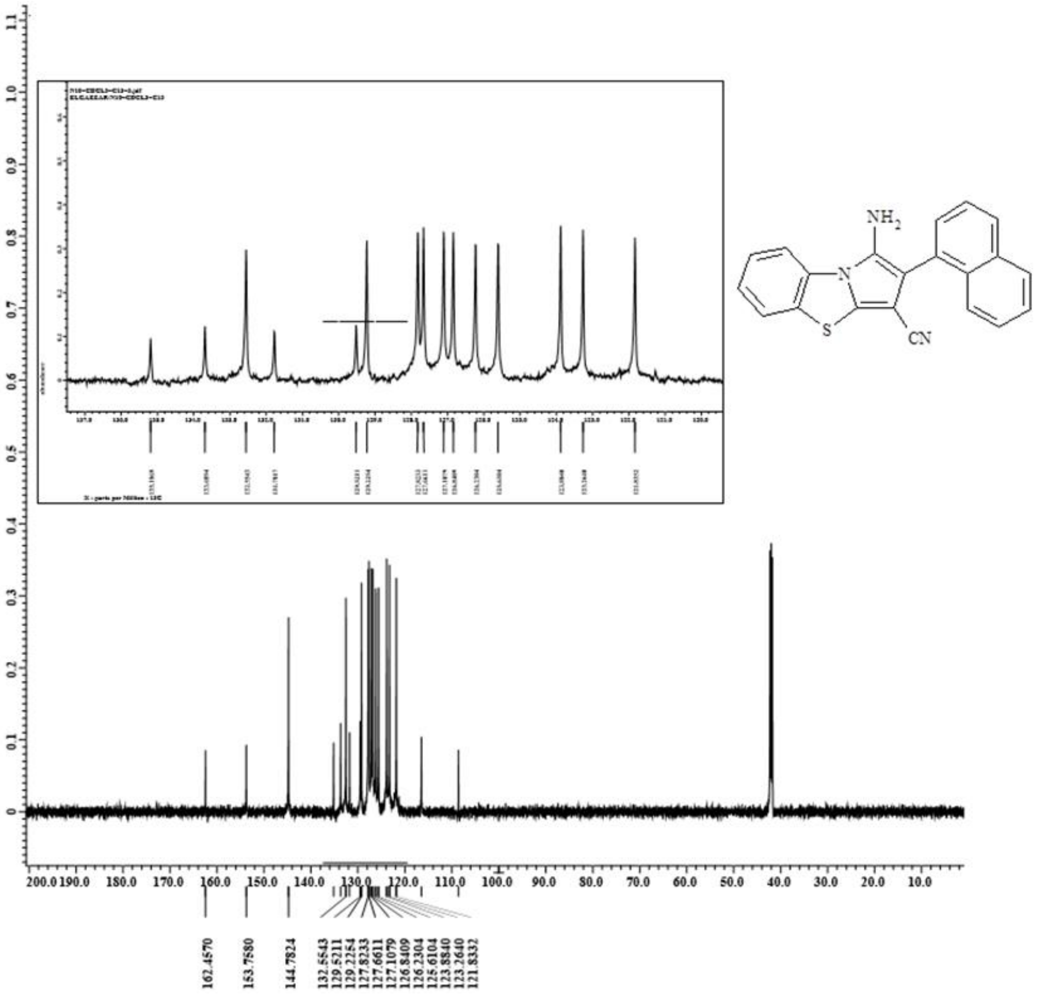
200.0 190.0 180.0 170.0 160.0 150.0 140.0 130.0 120.0 110.0 100.0 90.0 80.0 70.0 60.0 50.0 40.0 30.0 20.0 10.0
162.4570
153.7580
144.7824
132.5543
129.5211
129.2254
127.8233
127.6611
127.1079
126.8409
126.2304
125.6104
123.8840
123.2640
121.8332
NH_2
N
S
CN

^{1}H NMR and ^{13}C-NMR spectra of 1-amino-2-(4-fluorophenyl) pyrrolo[2,1-b][1,3]benzothiazole-3-carbonitrile (**9d**)

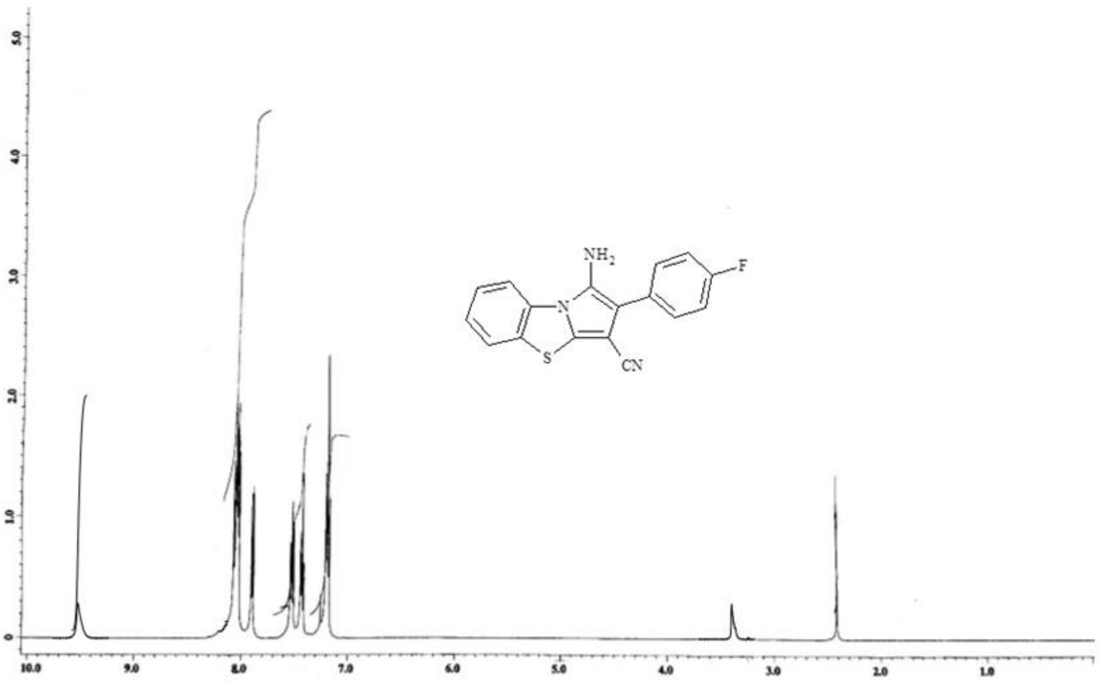

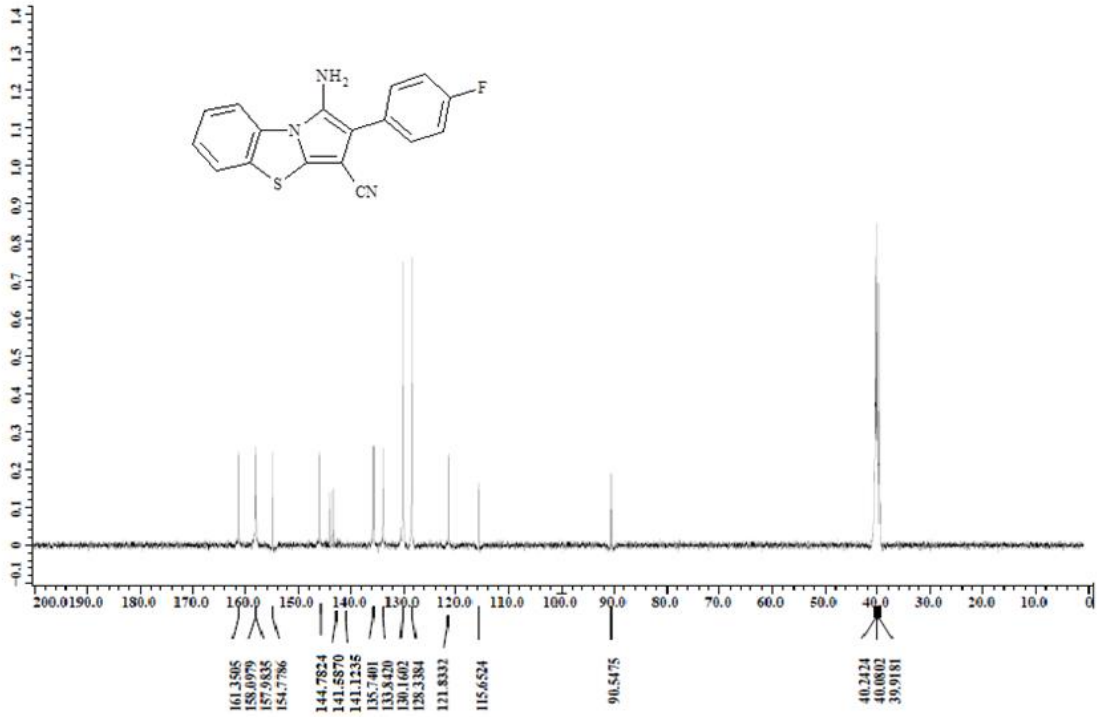

^{1}H NMR and ^{13}C-NMR spectra of 1-amino-2-(piperon-2-yl) pyrrolo[2,1-b] [1,3] benzothiazole-3-carbonitrile (**9e**)

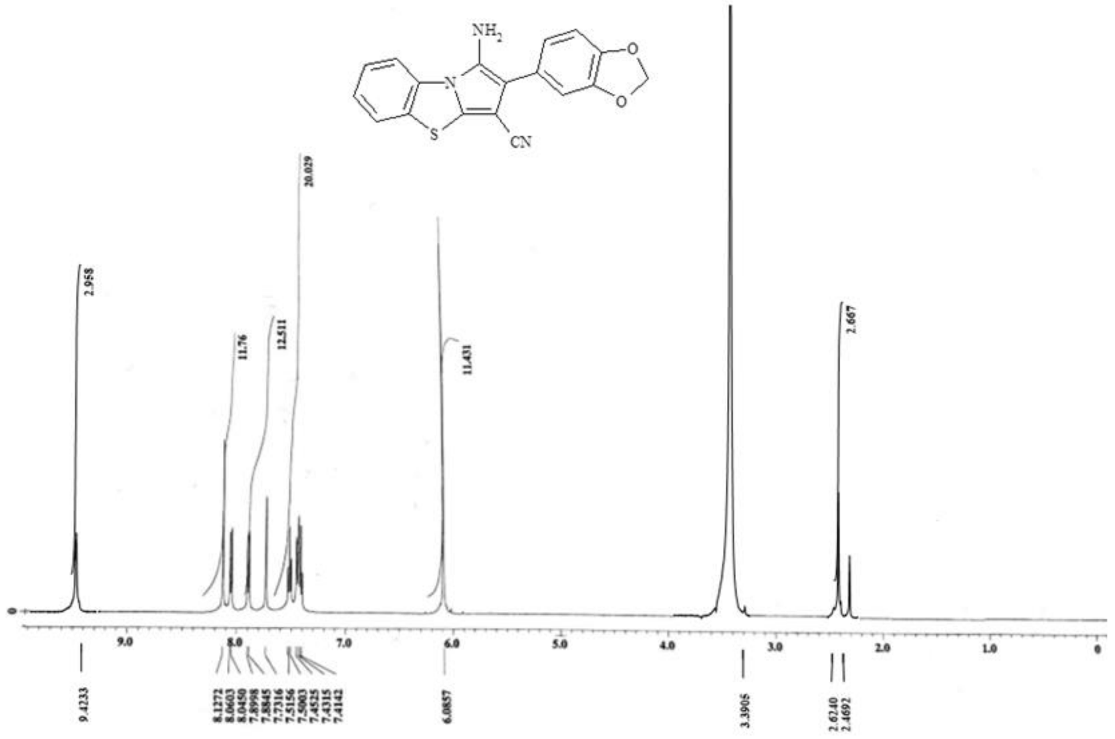

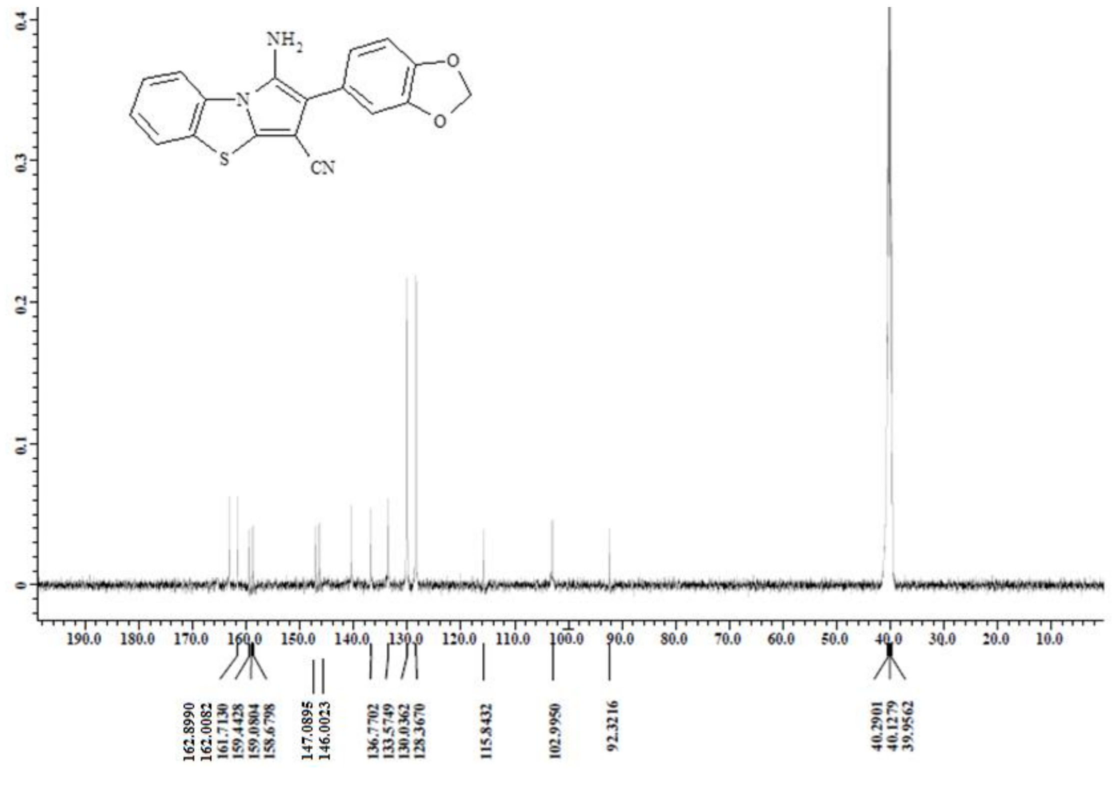

Supplement: Supplementary file 1 [file molecules-27-01246-s001.zip › molecules-1570475-Supplementary.pdf]
